# Analyzing the effect of ion binding to the membrane-surface on regulating the light-induced transthylakoid electric potential (ΔΨ_m_)

**DOI:** 10.3389/fpls.2022.945675

**Published:** 2022-07-28

**Authors:** Hui Lyu, Dušan Lazár

**Affiliations:** ^1^School of Biological Science and Agriculture, Qiannan Normal University for Nationalities, Duyun, China; ^2^Department of Biophysics, Faculty of Science, Palacký University, Olomouc, Czechia

**Keywords:** ions, thylakoid membrane, Donnan potential, diffusion potential, membrane potential, mathematical model

## Abstract

The transthylakoid membrane potential (ΔΨ_m_) is essential because it can drive the ATP synthesis through the CF_0_–CF_1_ type of ATP-synthase in chloroplasts as an energetic equivalent similar to ΔpH. In addition, a high fraction of proton motive force (PMF) stored as the ΔΨ_m_ component is physiologically important in the acclimation of photosynthesis to environmental stresses. It has been shown that ΔΨ_m_ is the sum of the Donnan potential difference (ΔΨ_dn_) and the diffusion potential difference (ΔΨ_d_). Specifically, ΔΨ_dn_, ΔΨ_d_, and ΔΨ_m_ are strongly associated with the ionic activities near the membrane surface, particularly, the extent of ion binding to the charged/neutral sites adjacent to the membrane surface. However, an in-depth analysis of the effect of altered cationic binding to the membrane surface on adjusting the transthylakoid electric potentials (ΔΨ_dn_, ΔΨ_d_, and ΔΨ_m_) is still missing. This lack of a mechanistic understanding is due to the experimental difficulty of closely observing cations binding to the membrane surface *in vivo*. In this work, a computer model was proposed to investigate the transthylakoid electric phenomena in the chloroplast focusing on the interaction between cations and the negative charges close to the membrane surface. By employing the model, we simulated the membrane potential and consequently, the measured ECS traces, proxing the ΔΨ_m_, were well described by the computing results on continuous illumination followed by a dark-adapted period. Moreover, the computing data clarified the components of transthylakoid membrane potential, unraveled the functional consequences of altered cationic attachment to the membrane surface on adjusting the transthylakoid electric potential, and further revealed the key role played by Donnan potential in regulating the energization of the thylakoid membrane. The current model for calculating electric potentials can function as a preliminary network for the further development into a more detailed theoretical model by which multiple important variables involved in photosynthesis can be explored.

## Introduction

Light-driven electron transport triggers the proton translocation across thylakoid membranes, because of which, a transmembrane proton motive force (PMF) is established. Transmembrane proton motive force consists of two components, i.e., a chemical (expressed as ΔpH) and an electrical component (expressed as ΔΨ_m_), which is defined by:


(1) $$\text{PMF}=\Delta \,\Psi_{m,i-o}\,\left(\frac{2.3\,\text{RT}}{\text{F}}\right)\,\Delta \,{\text{pH}}_{\text{o-i}}$$

where ΔΨ_*m,i*–*o*_ and ΔpH_*o*–*i*_ represent the electric potential difference and the pH difference across thylakoid membranes, respectively. They are calculated as outside (stroma) minus inside (lumen) for ΔpH and inside (lumen) minus outside (stroma) for ΔΨ_m_. The variables R, T, and F are the universal gas constant, the absolute temperature, and the Faraday constant, respectively. The PMF is the driving force for ATP synthesis facilitated by the CF_0_–CF_1_ type of ATP-synthase in chloroplasts. The two components of PMF are thermodynamically equivalent to drive ATP formation according to the chemiosmotic hypothesis (Mitchell, 1961, 1966, 2011). The ratio of PMF parsing into ΔΨ_m_ and ΔpH is a crucial quantity for reconciling the light use efficiency under rapid changes in environmental conditions (Davis et al., 2016, 2017; Li et al., 2021). More importantly, strong lumen acidification can initiate the process of non-photochemical quenching (NPQ) to protect photosystem II (PSII) from over-excitation by converting the excess light energy into heat (Li et al., 2009; Pinnola and Bassi, 2018). On the other hand, ΔΨ_m_ is physiologically essential because it not only functions to promote the ATP yield but also serves to regulate the electron transport reaction by affecting the midpoint redox potentials of electron acceptors and electron donors (Bulychev, 1984; Vredenberg and Bulychev, 2003; Davis et al., 2016). In addition, a high fraction of PMF stored as the ΔΨ_m_ component is particularly important during transitions from high to low light (Wang and Shikanai, 2019; Basso et al., 2020; Correa Galvis et al., 2020).

Moreover, ΔΨ_m_ is responsive to many factors including the composition of the bathing solution, the activity of proton pumping, the state of ion channels/transporters, and ionic activities near the membrane surface, which are themselves responsive to ΔΨ_m_ (Kinraide et al., 1998). It has been shown that ΔΨ_m_ is the sum of the Donnan potential difference (ΔΨ_dn_) and the diffusion potential difference (ΔΨ_d_) (Ohshima and Ohki, 1985; Ohshima and Kondo, 1987, 1988a,b, 1990). Specifically, Ψ_dn_ is the consequence of the interaction between solute ions in the contacting bulk-phase and the fixed charges arising from the protein ligands, since the thylakoid membrane is the lipo-protein system which carries the negative charges mainly due to carboxyl groups (probably glutamic and aspartic acid residues) of exposed segments of integral membrane protein constitutes (Barber, 1980b,^1982^). The specific value of the negative charges may be determined based on the microelectrophoresis studies and varies in terms of the plant species and physiological conditions within the same breed (Nakatani et al., 1978). The electrostatic ΔΨ_dn_ is often sufficiently negative to attract mobile divalent cations (e.g., Mg^2+^) more than mobile monovalent cations (e.g., K^+^, H^+^) and repel the mobile anions (e.g., Cl^–^, OH^–^). Previously, Siggel proposed a preliminary model to probe the role played by ΔΨ_dn_ during photosynthesis in a series of works (Siggel, 1981a,b,c). To date, the common neglect of ΔΨ_dn_ in a range of photosynthetic simulations probably reflects the computational complexity when all heterogeneous ions *in situ* together with the cations binding to the membrane surface are taken into account. Nevertheless, the existence of ΔΨ_dn_-induced electrical phenomena near the thylakoid membrane should be considered for an improved understanding of many aspects of the photosynthetic events in and around the thylakoid membrane (Barber, 1980b,^1982^; Kaña and Govindjee, 2016). An alternative approach to theoretically analyze the ion charge interaction adjacent to the membrane surface, termed the Gouy–Chapman double-layer potential, assumes that the thickness of membrane surface charge layer (d_s_) is zero and all membrane fixed charges are only located within the membrane surface (Barber, 1980a; Ohshima and Ohki, 1985; Ohshima and Kondo, 1990). This hypothesis may be unjustified because for a usual biological membrane, the d_s_ is commonly non-zero (Levine et al., 1983). Under this circumstance, the electrical potential in the region far inside the charge layer is practically equal to the Donnan potential (Ohshima and Kondo, 1988b).

The diffusion potential difference (ΔΨ_d_) is initiated by the light-induced electron-coupled proton translocation which causes the luminal acidification. This eventually triggers the proton efflux through the ATP-synthase and the transthylakoid ion movement *via* ion channels/transporters. Early electrophysiological experiments showed that thylakoid-harbored cation pores are permeable to K^+^ and Mg^2+^ (Pottosin and Schönknecht, 1996), while anion pores are permeable to Cl^–^ (Schönknecht et al., 1988). The recent molecular biological assays confirmed the existence of K^+^/H^+^ antiporter KEA3 (Kunz et al., 2014; Basso et al., 2020), the voltage-gated Cl^–^ channel VCCN1 (Herdean et al., 2016b), and the Cl^–^ channel CLCe (Herdean et al., 2016a). The co-function of the proton translocation and the channel/transporter mediated ion movement ultimately determine the ΔΨ_d_ which itself conversely impact the redistribution of ions and homeostasis of the stacked membranous system.

Numerous studies have concentrated majorly on the interrelationship between the chemical component of PMF (ΔpH) and its impact on photosynthetic processes. However, less effort was put on explorations of the electric component of PMF. Here, we proposed a photosynthetic model, which is an extension of a previously published version (Lyu and Lazar, 2017a,b), to determine the effect of ion attached to the thylakoid membrane surface on regulating ΔΨ_dn_, ΔΨ_d_, and ΔΨ_m_. Toward this end, the extension of model includes the determination of Ψ_dn_ and the corresponding change of quantities induced by Ψ_dn_. Taken together, our goal was to gain a mechanistic insight into these variations in the highly complex and dynamic network of photosynthetic processes.

## Materials and methods

**FIGURE 1:**
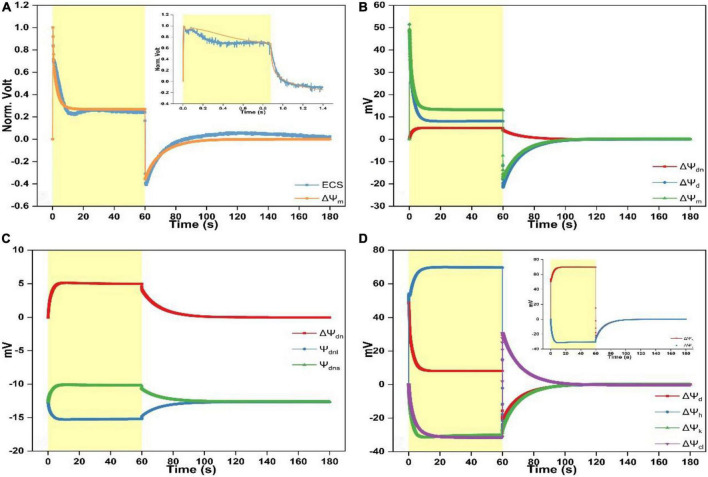
Time courses of electrochromic shift (ECS) spectra and simulated electric potentials. **(A)** Comparison of the simulated response of ΔΨ_m_ to the measured ΔA515–560-nm signal upon continuous illumination for 60 s followed by the dark adaptation for another 120 s. The insert depicts the comparison of the measured ΔA515–560-nm signal with the simulated response of ΔΨ_m_ upon continuous illumination for ∼1 s followed by the dark treatment for ∼0.5 s. **(B)** Simulated responses of ΔΨ_dn_, ΔΨ_d_, and ΔΨ_m_ upon continuous illumination for 60 s followed by the dark adaptation for another 120 s. **(C)** Simulated responses of Ψ_dnl_, Ψ_dns_, and ΔΨ_dn_ upon continuous illumination for 60 s followed by the dark adaptation for another 120 s. **(D)** Simulated responses of ΔΨ_d_, ΔΨ_h_, ΔΨ_k_, and ΔΨ_cl_ upon continuous illumination for 60 s followed by the dark adaptation for another 120 s. The insert depicts the simulated responses of ΔΨ_h_ and ΔΨ_k_. Light intensity for continuous light (duration 1 or 60 s) was 1,024 μmol photons m^– 2^ s^–1^.

Measurements were conducted with 10-day tobacco leaves (*Nicotiana tabacum*), grown in a growth chamber at 20°C on artificial soil composed of Perlit and Knop’s solution. The light regime was 8 h dark/16 h light (continuous white irradiation of 90 μmol photons m^–2^ s^–1^). Measured P515 signal was used as a proxy for ΔΨ_m_ (Klughammer et al., 2013). The P515 (ΔA515–550 nm) signal reflects the electrochromic shift (ECS) of absorption maximum of pigments (chlorophylls and carotenoids), which is proportional to ΔΨ_m_ across the thylakoid membrane (Witt, 1979; Vredenberg, 1981; Joliot and Joliot, 1989). ECS and P515 are used as synonyms in the text. The P515 signal measurements were done using Dual-PAM-100 system (Heinz Walz GmbH, Effeltrich, Germany) equipped with the P515/535 module. The system measures transmittance at 515 and 550 nm, and then their difference is assessed. The P515 signal was detected during continuous illumination of adaxial leaf side by white light of high intensity (1,024 μmol photons m^–2^ s^–1^). The measurements have been accomplished with seven different leaves and the typical curves are shown in Figure 1A and its inset. All samples were dark-adapted for 10 min before measurements.

## Description of the model

A comprehensive model of proton-coupled electron transport in and around photosynthetic thylakoid membranes has been proposed in our previous studies (Lyu and Lazar, 2017a,b). The model was made up of several modules including the linear and cyclic electron transport, the proton outflow through ATP-synthase, Calvin-Benson cycle and regulatory pathways. Moreover, the ion fluxes were calculated based on the modified Goldman–Hodgkin–Katz (GHK) equation (Van Kooten et al., 1986) and/or the transition state rate theory (TSRT) (Lyu and Lazar, 2017b). All quantitative values for model parameters are taken from literature and details of the model are given in our previous studies (Lyu and Lazar, 2017a,b). In the previous model, the ion–charge interaction was roughly speculated based on a preliminary calculation for the surface potential. In this work, we introduce the Donnan equilibrium potential to elaborate on the electrical phenomena adjacent to the thylakoid membrane-surface. Overall, Ψ_dn_ can regulate the distribution of ions close to the membrane surface causing the enrichment of cations and repulsion of anions; hence, adjusting the dynamics of channels/antiporters mediated ion fluxes and the proton efflux through ATP-synthase.

**SCHEME 1:**
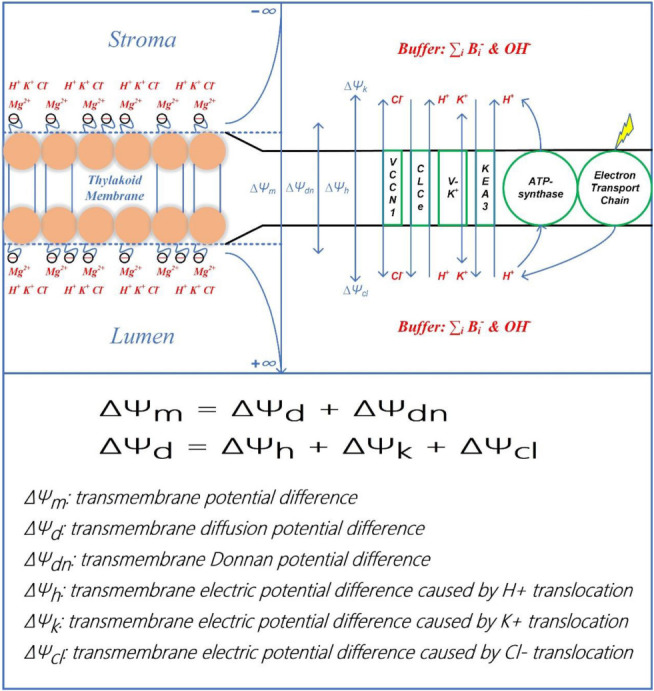
Schematic diagram of establishment and classification of transthylakoid electric potential. The upper panel presents the establishment of ΔΨ_m_ and its components. The onset of electron transport as illumination commences is coupled to the proton translocation from the stroma into the lumen. The proton motive force is thus established due to the acidification of the lumen and the alkalization of the stroma, which is ultimately used to drive the ATP synthesis through the CF_0_–CF_1_ type of ATP-synthase in chloroplasts. In response to the environmental conditions, the proton motive force is partitioned into its two components (ΔpH and ΔΨ_m_) *via* ion channels/antiporters, i.e., the voltage-gated K^+^ channel V–K^+^, the K^+^/H^+^ antiporter KEA3, the voltage-gated Cl^–^ channel VCCN1, and the Cl^–^ channel CLCe. The resultant effect of ion redistribution causes the formation of the diffusion potential difference (ΔΨ_d_) which includes three components, i.e., ΔΨ_h_, ΔΨ_k_, and ΔΨ_cl_. It can be observed that both surfaces of the thylakoid membrane carry fixed negative charges whilst the negative charge density on the luminal side is more significant than the stromal side. To equilibrate the initial transthylakoid Donnan potential in darkness, the fixed negative charge on either membrane-surface is equally assigned the value of −0.035 C m^–2^. Besides, Donnan potential difference (ΔΨ_dn_) is established between the membrane-surface and the electrolyte solution. The electrostatic ΔΨ_dn_ is often sufficiently negative to attract mobile divalent cations (e.g., Mg^2+^) more than mobile monovalent cations (e.g., K^+^, H^+^) and repel mobile anions (e.g., Cl^–^, OH^–^). The enriched cations (Mg^2+^, K^+^, and H^+^) can also bind to negative charge sites and/or neutral sites, ultimately altering the membrane-surface negativity. In the lower panel, the calculating equation for ΔΨ_m_ and the definition for all components of ΔΨ_m_ are clarified.

To clarify, the overall concept of the model is presented in Scheme 1. The onset of the electron transport is coupled to the proton translocation from the stroma into the lumen. The PMF is thus established and then consumed to drive the ATP synthesis through the CF_0_–CF_1_ type of ATP-synthase in chloroplasts. The early electrophoresis findings show that the negative charge density (∼−0.035 C/m^2^) on the luminal side is higher than on the stromal side (∼−0.025 C/m^2^) (Barber, 1982). Nonetheless, to equilibrate the initial transthylakoid Donnan potential in darkness, the fixed negative charge on either membrane surface is evenly assigned the value of −0.035 C/m^2^ (Barber, 1982). Several early physiological measurements also showed a rise, upon illumination, of Mg^2+^ concentration in the stroma by 1–5 mM (Krause, 1977; Portis, 1981) and it was speculated that the change of stromal Mg^2+^ concentration may affect the thylakoid stacking and hence the efficiency of energy transfer between photosystems I and II (Barber et al., 1977), but the relative contribution of Mg^2+^ ions remains a matter of discussion because Tester and Blatt (1989) also reported that there was no Mg^2+^ current in a direct measurement of ion movement by fusing spinach thylakoid vesicles into planar lipid bilayers (Tester and Blatt, 1989). It seems that the Mg^2+^ flux presented in the literature vary greatly and is highly impacted by assay conditions. Therefore, it has been suggested that the Mg^2+^ flux may only play a small part in balancing the light-driven H^+^ movements (Tester and Blatt, 1989) and it has also been suggested that under physiological conditions the current through the cation selective channel is mainly carried by K^+^ ions (Pottosin and Schönknecht, 1996). Furthermore, the Mg^2+^ related channels/antiporters in thylakoid membranes have not been identified so far by modern molecular studies. Therefore, it would be reasonable to only consider K^+^ and Cl^–^ as the main ions penetrating the membrane to modify the electric potentials, which has been suggested as well in a recently published model to *in silico* study the partitioning of PMF upon various light conditions (Li et al., 2021). To follow up, ions of K^+^ and Cl^–^ are redistributed to partition ΔΨ_m_ into ΔΨ_d_ and ΔΨ_dn_ *via* ion channels/antiporters, namely, the voltage-gated K^+^ channel V–K^+^, the K^+^/H^+^ antiporter KEA3, the voltage-gated Cl^–^ channel VCCN1, and the Cl^–^ channel CLCe. The resultant effect of ion redistribution causes the formation of the diffusion potential difference (ΔΨ_d_) which consists of three components, i.e., ΔΨ_h_, ΔΨ_k_, and ΔΨ_cl_. The concepts and terms necessary for the understanding of the results and discussion are described below and detailed description of all computational segments of the model is provided in Supplementary Material.

### The calculation of electrical potential difference

As presented above, ΔΨ_m_ is responsive to the composition of the contacting solution, the activity of proton pumping, the state of ion channels/antiporters, and electrical characteristics near the membrane surface. In brief, ΔΨ_m_ and ΔΨ_d_ can be calculated by (see Scheme 1):


(2) $$\Delta \,\Psi_{\text{m}}=\Delta \,\Psi_{\text{d}}+\Delta \,\Psi_{\text{dn}}$$

(3) $$\Delta \,\Psi_{\text{d}}=\Delta \,\Psi_{\text{h}}+\Delta \,\Psi_{\text{k}}+\Delta \,\Psi_{\text{cl}}$$

Where ΔΨ_m_ equals to the sum of ΔΨ_d_ and ΔΨ_d*n*_; ΔΨ_h_, ΔΨ_k_, and ΔΨ_cl_ sum up to form ΔΨ_d_; ΔΨ_d_, ΔΨ_dn_, ΔΨ_h_, ΔΨ_k_, and ΔΨ_cl_ are caused, respectively, by the light-induced transmembrane redistribution of bulk electrolytes, by the interplay of negative thylakoid-membrane surface charges and the bathing solute ions, by the transmembrane H^+^ movement and H^+^ released from oxygen evolving complex (OEC), by the transmembrane K^+^ movement, and by the transmembrane Cl^–^ movement. More information on calculation of ΔΨ_d_ can be obtained in our previous works (Lyu and Lazar, 2017a,b).

### The Donnan potential determination involving the ion attachment to the membrane surface

The Donnan potential is the electrical potential at which the electroneutrality of the entire system is reached (Ohshima and Kondo, 1990). It occurs due to the excess of impermeant anions (e.g., charged macromolecules and/or organic acids) sequestered in a living cell. This nonzero potential changes the concentrations of solute ions near the membrane away from their values in the bulk solution (Gillespie and Eisenberg, 2001), which can be determined from:


(4) $$\sum_{\text{i}=1}^{\text{N}}{\text{z}}_{\text{i}}\,{\text{C}}_{\text{i}}^{\infty }\,\text{exp}\,\left(-\frac{{\text{z}}_{\text{i}}\,\text{F}\,\Psi_{\text{dn}}}{\text{RT}}\right)+\frac{\rho }{\text{F}}\times \text{ro}=0$$

where *z*_*i*_ is the valence of the *i*th ion (*i* = 1, 2, …, *N*), *C*_*i*_^∞^ is the bulk concentration of the *i*th ion, F is the Faraday constant, R is the universal gas constant, T is the absolute temperature, ρ is the density of the non-neutralized membrane-fixed negative charges, and ro is the ratio of surface-area to volume, which is assigned the value of 10^6^ cm^–1^ for thylakoid (Van Kooten et al., 1986).

Ions binding to the membrane-surface are incorporated by altering ρ in Eq. (4). Here, ρ depends in part on intrinsic surface charge density and also includes those solute ions that bind to the membrane surface. In this study, the model takes into account 1:1 binding of cations to a negatively charged site ([R^–^]) and a neutral site ([P^0^]) as described in the following reactions:


(5) $$R^{-}+I_{i}^{z_{i}}\to {\left(R\,I_{i}\right)}^{z_{i}-1}$$

(6) $$R^{-}\times I_{i}^{z_{i}}\times K_{c\,i}={\left(R\,I_{i}\right)}^{z_{i}-1}$$

and


(7) $${\text{P}}^{0}+{\text{I}}_{\text{i}}^{{\text{z}}_{\text{i}}}\to {\left({\text{PI}}_{\text{i}}\right)}^{{\text{z}}_{\text{i}}}$$

(8) $${\text{P}}^{0}\times {\text{I}}_{\text{i}}^{{\text{z}}_{\text{i}}}\times {\text{K}}_{\text{pi}}={\left({\text{PI}}_{\text{i}}\right)}^{{\text{z}}_{\text{i}}}$$

and


(9) $${\text{I}}_{\text{i}}^{{\text{z}}_{\text{i}}}={\text{C}}_{\text{i}}\times \text{exp}\,\left(-\frac{{\text{z}}_{\text{i}}\,\text{F}\,\Psi_{\text{dn}}}{\text{RT}}\right)$$

Thus, ρ can be computed from Eqs (4) to (8) as follows:


(10) $$\frac{\rho }{\text{F}}\times \text{ro}=-\left[{\text{R}}^{-}\right]+\sum_{\text{i}=1}^{\text{N}}\left({\text{z}}_{\text{i}}-1\right)+\left[{\left({\text{RI}}_{\text{i}}\right)}^{{\text{z}}_{\text{i}}-1}\right]+\sum_{\text{i}=1}^{\text{N}}{\text{z}}_{\text{i}}\,\left[{\left({\text{PI}}_{\text{i}}\right)}^{{\text{z}}_{\text{i}}}\right]$$

where *z*_*i*_ is the valence of the *i*th ion (*i* = 1, 2, …, *N*), I_*i*_*^zi^* is the Ψ_dn_-induced concentration of the ith ion close to the membrane surface, [R^–^] and [P^0^] denote concentrations of the negatively charged sites and the neutral sites ([R^–^] equals to 3.63 × 10^–5^ mol/cm^3^ reflects [SC] which was set at −0.035 C/m^2^; P^0^ reflects [SP] and was assigned 8-fold of [R^–^]), [(RI_*i*_)^*zi*–1^] and [(PI_*i*_)*^zi^*] denote product concentrations of the *i*th ion binding to the negatively charged site and the neutral site. For cations binding to the negative charge sites, both divalent (Mg^2+^) and monovalent species (K^+^ and H^+^) are considered in simulations. However, it can be observed from Eq. (10) that the concentration of negative charges remains unaffected provided a neutralized pair is randomly created for the monovalent cations binding to the negative charges. These formulas were initially proposed to investigate the rhizotoxicity of heavy metals binding to the root cell membrane (Kinraide et al., 1998). Unless stated otherwise, all theoretical details and parameter values involved in the formulations are presented in Supplementary Material.

## Results

**FIGURE 2:**
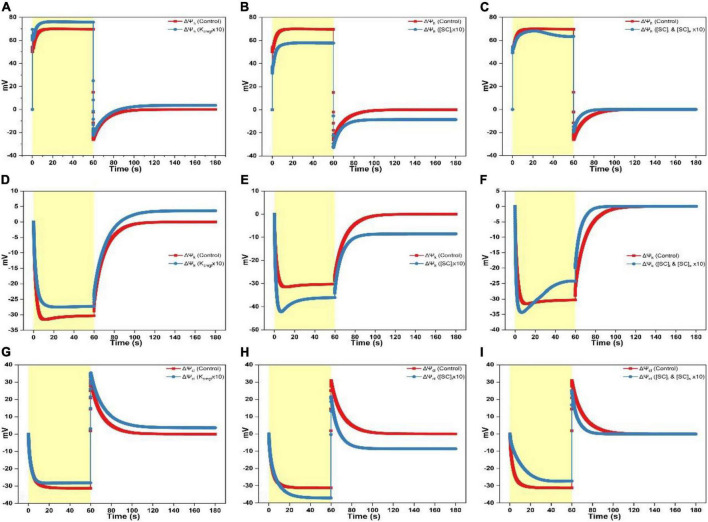
Simulated responses of ΔΨ_h_, ΔΨ_k_, and ΔΨ_cl_ on assigning different values for adjustable parameters. **(A)** Simulated responses of ΔΨ_h_ by adjusting K_cmgl_ 1- and 10-fold of the controlled value. **(B)** Simulated responses of ΔΨ_h_ by adjusting [SC]_*l*_ 1- and 10-fold of the controlled value. **(C)** Simulated responses of ΔΨ_h_ by adjusting [SC]_*l*_ and [SC]_s_ 1- and 10-fold of the controlled value. **(D)** Simulated responses of ΔΨ_k_ by adjusting K_cmgl_ 1- and 10-fold of the controlled value. **(E)** Simulated responses of ΔΨ_k_ by adjusting [SC]_*l*_ 1- and 10-fold of the controlled value. **(F)** Simulated responses of ΔΨ_k_ by adjusting [SC]_*l*_ and [SC]_s_ 1- and 10-fold of the controlled value. **(G)** Simulated responses of ΔΨ_cl_ by adjusting K_cmgl_ 1- and 10-fold of the controlled value. **(H)** Simulated responses of ΔΨ_cl_ by adjusting [SC]_*l*_ 1- and 10-fold of the controlled value. **(I)** Simulated responses of ΔΨ_cl_ by adjusting [SC]_*l*_ and [SC]_s_ 1- and 10-fold of the controlled value.

Following continuous illumination for 60 s and the ensuing 120 s in darkness, the ECS kinetics is in accordance with the previously reported measurements (Kramer and Sacksteder, 1998; Johnson and Ruban, 2014). Specifically, upon switching on the light, the ECS kinetics shows a sharp rise before decaying to ∼30% of its initial amplitude and then rising again till saturating within ∼20 s. When the light is switched off, the ECS signal shows a sharp fall before returning to the steady-state a bit higher above the dark baseline (Figure 1A). Overall, the ECS kinetics was well described by the modeled ΔΨ_m_ whereas the simulation lacks the shallow dip positioned during ∼10 to ∼30 s, and the ECS kinetics as illumination ceases appears to relax a bit faster than the modeled curve. According to Johnson and Ruban (2014), the transient dip and the following changes leading to the steady-state before illumination ceases is the slow phase of ECS, which is not caused by ΔΨ_m_ but the formation of qE-related absorption change. However, Kramer et al. suggested that a significant fraction of PMF in chloroplasts can be stored as ΔΨ_m_ under the steady-state conditions (Kramer and Sacksteder, 1998; Kramer et al., 1999; Cruz et al., 2001, 2005; Davis et al., 2017; Kanazawa et al., 2017). Our simulations show that the lasting ECS trace is partly contributed by ΔΨ_dn_ (see Figure 1B), and the dip may be related to K^+^ redistribution induced by the alteration of abundance of membrane surface charges on luminal surface or both surfaces ([SC]_*l*_ or [SC]_*l*_ and [SC]_s_) (see Figures 2E,F). The insert of Figure 1A shows the comparison between the modeled ΔΨ_m_ and the ECS signal upon illumination for ∼1 s followed by a 0.5-s period of darkness. The measured ECS curve shows a bimodal pattern as illumination commences before decaying to a plateau. Upon switching off the light, the curve shows an exponential decay till reaching a steady-level largely below the dark baseline, in line with the measurements previously reported in the literature (Bulychev, 1984; Vredenberg, 1997; Bulychev and Vredenberg, 1999). The modeled ΔΨ_m_ can reproduce several features of the measured curve, e.g., the bimodal pattern, the timing of the first peak and the transient dip after the first peak, and the decaying kinetics in the darkness. However, the second peak of simulation appears to be less prominent than in the ECS signal, which may be related to the proton translocation induced by the alteration of abundance of membrane-surface charges on both membrane sides ([SC]_*l*_ and [SC]_s_) (see Figure 2C). Figure 1B shows that ΔΨ_dn_ and ΔΨ_d_ sum up to form ΔΨ_m_ in which ∼40% amplitude of the steady-state ΔΨ_m_ is contributed by the formation of ΔΨ_dn_ (∼5 mV). The simulated ΔΨ_dn_, which is the difference of Ψ_dns_ minus Ψ_dnl_, is shown in Figure 1C. The negativity of Ψ_dnl_ is increased because of the light-driven cation efflux into the stroma (K^+^ out and H^+^ out) and anion influx into the lumen (Cl^–^ in). The Donnan equilibrium potential in either compartment is usually less than ∼−20 mV (Sperelakis, 2012) and apparently our results are approaching to this value.

The separation of the simulated ΔΨ_d_ into three components, namely, the electric potential difference caused by H^+^ translocation (ΔΨ_h_), by K^+^ translocation (ΔΨ_k_), and by Cl^–^ translocation (ΔΨ_cl_) is shown in Figure 1D. As the light is switched on, the ΔΨ_h_ trace shows a sharp rise followed by a dip before rising again till reaching a steady-state. In contrast, ΔΨ_k_ and ΔΨ_cl_, as the counterpart of ΔΨ_h_, both decreased although ΔΨ_k_ decreased initially faster than ΔΨ_cl_ till reaching a negative steady-level (Figure 1D). As the light is switched off, the ΔΨ_h_ trace contains a sharp fall till the inverted ΔΨ_h_ attains the negative maximum and then followed by a slow relaxation returning to the dark baseline which is attributable to the leaky proton influx into the lumen (Supplementary Figures 3A,B). The inverted ΔΨ_h_ would suddenly force Cl^–^ back into the stroma till ΔΨ_cl_ is inverted to the positive maximum before relaxing to the dark baseline (Figure 1D). Simultaneously, the K^+^ flux out of the lumen will be decelerated due to the inverted ΔΨ_h_ till ΔΨ_k_ converges to the dark baseline. Notably, the ΔΨ_k_ relaxing trace during the dark phase is overlapped by the ΔΨ_h_ trace (insert of Figure 1D). This is understandable since the K^+^ efflux is mediated by the V–K^+^ channel which is strictly voltage dependent.

**FIGURE 3:**
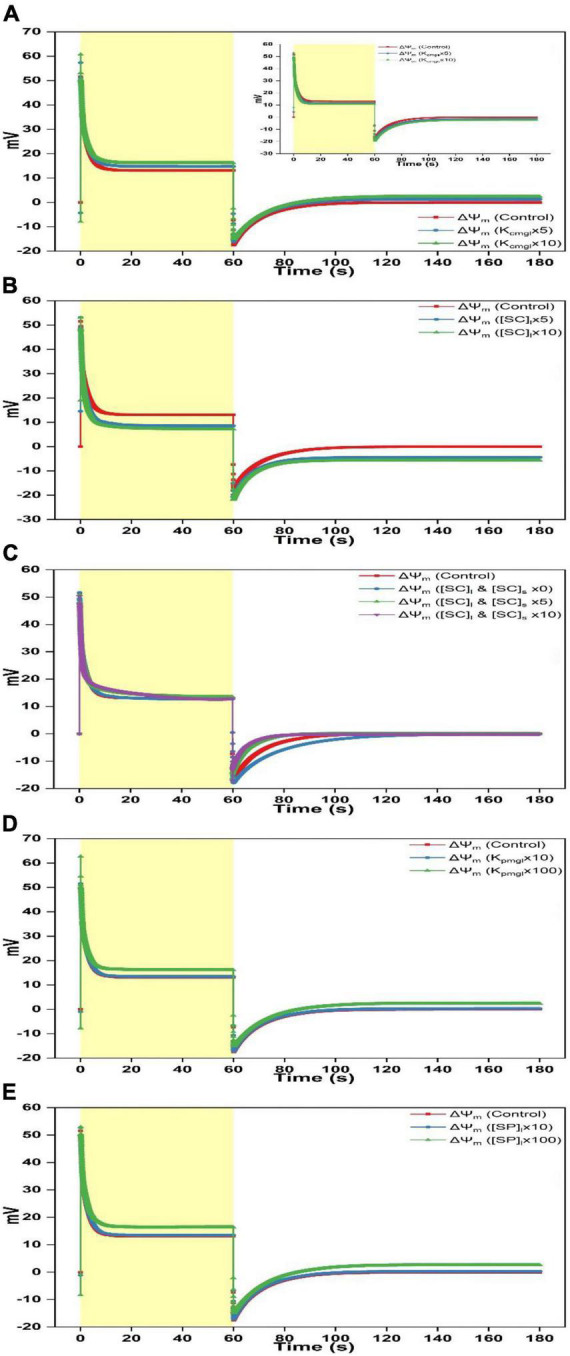
Simulated responses of ΔΨ_m_ on assigning different values for adjustable parameters. **(A)** Simulated responses of ΔΨ_m_ by adjusting K_cmgl_ 1-, 5-, and 10-fold of the controlled value. The insert illustrates simulated responses of ΔΨ_m_ by adjusting K_cmgs_ 1-, 5-, and 10-fold of the controlled value. **(B)** Simulated responses of ΔΨ_m_ by adjusting [SC]_*l*_ 1-, 5-, and 10-fold of the controlled value. **(C)** Simulated responses of ΔΨ_m_ by adjusting [SC]_*l*_ and [SC]_s_ 0-, 1-, 5-, and 10-fold of the controlled value. **(D)** Simulated responses of ΔΨ_m_ by adjusting K_pmgl_ 1-, 10-, and 100-fold of the controlled value. **(E)** Simulated responses of ΔΨ_m_ by adjusting [SP]_*l*_ 1-, 10-, and 100-fold of the controlled value.

**FIGURE 4:**
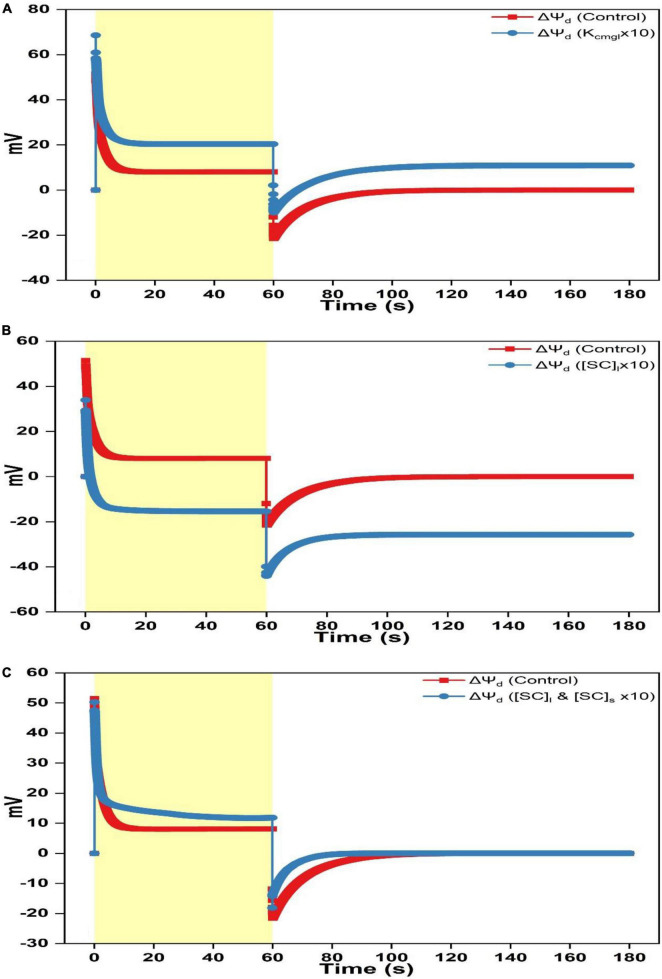
Simulated responses of ΔΨ_d_ on assigning different values for adjustable parameters. **(A)** Simulated responses of ΔΨ_d_ by adjusting K_cmgl_ 1- and 10-fold of the controlled value. **(B)** Simulated responses of ΔΨ_d_ by adjusting [SC]_*l*_ 1- and 10-fold of the controlled value. **(C)** Simulated responses of ΔΨ_d_ by adjusting [SC]_*l*_ and [SC]_s_ 1- and 10-fold of the controlled value.

**FIGURE 5:**
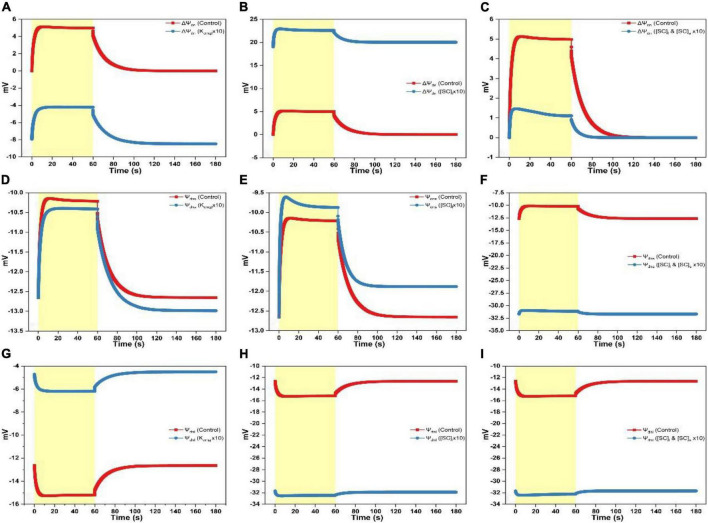
Simulated responses of Ψ_dnl_, Ψ_dns_, and ΔΨ_dn_ on assigning different values for adjustable parameters. **(A)** Simulated responses of ΔΨ_dn_ by adjusting K_cmgl_ 1- and 10-fold of the controlled value. **(B)** Simulated responses of ΔΨ_dn_ by adjusting [SC]_*l*_ 1- and 10-fold of the controlled value. **(C)** Simulated responses of ΔΨ_dn_ by adjusting [SC]_*l*_ and [SC]_s_ 1- and 10-fold of the controlled value. **(D)** Simulated responses of Ψ_dns_ by adjusting K_cmgl_ 1- and 10-fold of the controlled value. **(E)** Simulated responses of Ψ_dns_ by adjusting [SC]_*l*_ 1- and 10-fold of the controlled value. **(F)** Simulated responses of Ψ_dns_ by adjusting [SC]_*l*_ and [SC]_s_ 1- and 10-fold of the controlled value. **(G)** Simulated responses of Ψ_dnl_ by adjusting K_cmgl_ 1- and 10-fold of the controlled value. **(H)** Simulated responses of Ψ_dnl_ by adjusting [SC]_*l*_ 1- and 10-fold of the controlled value. **(I)** Simulated responses of Ψ_dnl_ by adjusting [SC]_*l*_ and [SC]_s_ 1- and 10-fold of the controlled value.

To investigate the effect of ion attached to the membrane surface on regulating ΔΨ_dn_, ΔΨ_d_, and ΔΨ_m_, the controlling parameters that affect electric characteristics close to the membrane surface are adjusted including the binding constant of Mg^2+^ attached to the negative charge sites on both surfaces (K_cmgl_ and K_cmgs_), the binding constants of Mg^2+^, K^+^, and H^+^ attached to the neutral sites on both surfaces (K_pmgl_, K_pmgs_, K_pkl_, K_pks_, K_phl_, and K_phs_), the density of the negative charge site on both surfaces ([SC]_*l*_ and [SC]_s_), and the density of the neutral site on both surfaces ([SP]_*l*_ and [SP]_s_). The change of these parameters or their compositions can take place near the luminal surface or the stromal surface or both. Since the variation of parameters near the stromal surface leads to the kinetics, which is sorted in a reverse order in comparison to the variation on single luminal surface (e.g., see insert of Figure 3A). Here, we consider the change of parameters occurring only on luminal surface or both surfaces. On luminal side, we have conducted simulations for 36 times by adjusting [SC]_*l*_ (control, 5-, and 10-fold), [SP]_*l*_ (0-fold, control, 10-, and 100-fold, the same change for the parameter composition followed), K_cmgl_, K_pmgl_, K_pkl_, K_phl_, [K_pmgl_K_pkl_], [K_pmgl_K_phl_], [K_pkl_K_phl_], and [K_pmgl_K_pkl_K_phl_], respectively. Similarly, a total of 37 simulations have been conducted by simultaneously adjusting parameters on both surfaces to the same extend as presented above except for the condition of [SC]_*l*_ and [SC]_s_, which adds one more trial (0-fold). Simply diminishing [SC]_*l*_ to zero only on luminal face will cause the significant deviation of initial quantitative values for key variables from the equilibrium which ultimately cause the breakdown of the model operation, we thus explore the impact of negative surface charges by concurrently increasing [SC]_*l*_ and [SC]_s_ on both sides. For all of the 0-fold changes related to [SP]_*l*_/[SP]_s_, K_cmgl_/K_cmgs_, K_pmgl_/K_pmgs_, K_pkl_/K_pks_, and K_phl_/K_phs_, the modeled traces rarely show the difference to the standard curves. Therefore, only the controlled traces will be presented in figures. In brief, all simulations (73 trials) that show the obvious alteration compared to the controlled simulation are shown in Figure 3. These alterations can basically be summarized into the following three types: (i), the ΔΨ_m_ curve induced by the increased K_cmgl_ was characterized as a more negative starting point, a higher peak, a faster decay till reaching the steady-state during the light phase as well as a more rapid fall followed by an accelerated relaxation in the darkness (Figure 3A). This pattern was also reproduced in Figures 3D,E *via* 100-fold increasing K_cmgl_ or [SP]_*l*_ whereas the variation appears to be less prominent for the change of 10-fold of the controlled value (Figures 3D,E). The variation for the ΔΨ_m_ curve was due to an upleveled ΔΨ_d_ (Figure 4A) and a crossover of ΔΨ_dn_ from the positive to the negative (Figure 5A) which is mainly contributed by a less negative Ψ_dnl_ (Figure 5G) whereas Ψ_dns_ is slightly affected (Figure 5D). (ii), the ΔΨ_m_ curve for the increased [SC]_*l*_ (Figure 3B) contains the kinetic behavior antiparallel to the curve caused by the increased K_cmgl_. This was due to a significantly decreased ΔΨ_d_ (Figure 4B) and a more positive ΔΨ_dn_ (Figure 5B) majorly contributed by an enormously negative Ψ_dnl_ (Figure 5H); (iii), the ΔΨ_m_ kinetics shows a different pattern induced by concurrently increasing [SC]_*l*_ and [SC]_s_ (Figure 3C), which shows a sharp rise as the light is switched on before experiencing a biphasic decay (a rapid decay followed by a slower decay) till reaching a steady-level. When the light is switched off, the ΔΨ_m_ kinetics shows a sharp fall below the dark baseline before returning to the steady-state much faster than in the controlled trace, whereas a much slower relaxation is visible for 0-fold of the controlled values (Figure 3C).

Overall, ΔΨ_dn_ and Ψ_dns_ progress in the same trend, i.e., the curve shows a rapid rise as illumination commences before attaining the steady-level followed by an exponential decay till the steady-state is reached as the light is switched off (Figures 5A–F). In contrast, the Ψ_dnl_ trace exhibits an opposite behavior which shows a rapid fall before attaining the steady-level followed by a slower rise till relaxing to the steady-state as the light is switched off (Figures 5G–I). The ΔΨ_dn_ curve was transferred to a more negative level by increasing K_cmgl_ (Figure 5A). This was majorly contributed by a smaller Ψ_dnl_ produced (less negative, Figure 5G). Intriguingly, increasing K_cmgl_ will cause a slight decrease of Ψ_dns_ (Figure 5D) which may be associated with the ion redistribution caused by various ionic fluxes. When increasing [SC]_*l*_, the ΔΨ_dn_ trace was transferred to a more positive level (Figure 5B) due to a much larger Ψ_dnl_ (more negative, Figure 5H) together with a slight increase of Ψ_dns_ produced (Figure 5E). When concurrently increasing [SC]_*l*_ and [SC]_s_, the ΔΨ_dn_ trace was transferred to a much smaller positive level (Figure 5C) due to a much larger Ψ_dnl_ (more negative as shown in Figure 5I similar as induced by individually increasing [SC]_*l*_ in Figure 5I) together with a significant upregulation of Ψ_dns_ produced (more negative as shown in Figure 5F for Ψ_dnl_).

When K_cmgl_ or [SC]_*l*_ is increased, the ΔΨ_d_ curve is as a whole upshifted to a more positive level (Figure 4A) or downshifted to a more negative level (Figure 4B) with an extent for the latter is a bit larger than the former. When concurrently increasing [SC]_*l*_ and [SC]_s_, the ΔΨ_d_ curve (Figure 4C) decays in a biphasic pattern till saturating in the light phase and shows an accelerated rise returning to the steady-state in the darkness.

When increasing K_cmgl_ 10-fold of the controlled value, the ΔΨ_h_ curve reaches a higher peak (∼70 mV) followed by a more prominent dip as the light is switched on till saturating to a larger steady-level (∼75 mV) (Figure 2A). As shutting off the light, the ΔΨ_h_ curve shows a rapid fall followed by an accelerated rise till reaching the steady-state slightly above the dark baseline (Figure 2A). The steady-state value of the ΔΨ_k_ curve for 10-fold condition is smaller (less negative) than the standard curve during the light phase (Figure 2D) and a more speeded relaxation occurs until reaching the steady-state significantly above the dark baseline as shutting off the light (Figure 2D). The steady-state value of the ΔΨ_cl_ curve for 10-fold condition is a bit smaller (less negative) than the standard trace during the light phase (Figure 2G). As switching off the light, the ΔΨ_cl_ curve shows a sharp rise followed by a decelerated exponential decay till reaching a steady-state moderately above the dark baseline than the controlled curve (Figure 2G).

When increasing [SC]_*l*_ 10-fold of the controlled value, the ΔΨ_h_ curve peaks at a lower level followed by a nearly diminished dip as switching on the light till saturating to a much smaller steady-level (∼58 mV) (Figure 2B). As shutting off the light, the ΔΨ_h_ curve shows a rapid fall followed by a faster rise till reaching the steady-state enormously below the dark baseline (Figure 2B). Upon the 10-fold increase of [SC]_*l*_, the ΔΨ_k_ curve shows a sharp fall followed by an acute dip till attaining the steady-level much higher (more negative) than the standard trace during the light phase (Figure 2E). As shutting off the light, an accelerated relaxation occurs until reaching the steady-state ∼10 mV below the dark baseline (Figure 2E). Similarly, upon the 10-fold increase of [SC]_*l*_, the ΔΨ_cl_ curve shows a slower exponential decay till attaining the steady-level much higher (more negative) than the controlled trace during the light phase (more negative, Figure 2H). As shutting off the light, the ΔΨ_cl_ curve shows a sharp rise reaching a smaller peak level (∼20 mV) followed by a much faster decay till reaching the steady-state ∼10 mV below the dark baseline (Figure 2H).

When concurrently increasing [SC]_*l*_ and [SC]_s_ 10-fold of the controlled value, the ΔΨ_h_ curve reaches the peak followed by a fast rise to a maximum at ∼25 s before decaying to a slightly smaller steady-level (∼62 mV) than the standard curve (Figure 2C). As switching off the light, the ΔΨ_h_ curve shows a sharp fall followed by a faster rise till reaching the dark baseline (Figure 2C). The ΔΨ_k_ curve upon the 10-fold increase of [SC]_*l*_ and [SC]_s_ shows a sharp fall to about ∼35 mV followed by a fast rise till leveling toward a smaller steady-level (∼25 mV) (Figure 2F). As switching off the light, a faster relaxation occurs until converging to the dark baseline as the controlled trace (Figure 2F). Upon concurrently increasing [SC]_*l*_ and [SC]_s_ 10-fold of the controlled value, the ΔΨ_cl_ curve shows a slower exponential decay till attaining the steady-level smaller (less negative) than the controlled curve in the light phase (Figure 2I). As switching off the light, the ΔΨ_cl_ curve shows a sharp rise peaking at a smaller level (∼25 mV) followed by an accelerated decay till converging to the dark baseline concomitant with the standard trace (Figure 2I).

## Discussion

### Specific roles of ion binding to the membrane-surface on regulating ΔΨ_dn_, ΔΨ_d_, and ΔΨ_m_

To investigate specific roles of ion binding to the membrane-surface on regulating ΔΨ_dn_, ΔΨ_d_, and ΔΨ_m_, controlling parameters which affect the electric characteristics adjacent to the membrane surface (i.e., K_cmgl_, K_cmgs_, K_pmgl_, K_*p*mgs_, K_pkl_, K_pks_, K_ph*l*_, K_chs_, [SC]_l_, [SC]_s_, [SP]_*l*_, and [SP]_s_) were adjusted under various scenarios to conduct *in silico* experiments. Computing findings reveal that approximate three types of variations induced by the adjustable parameters may be derived as follows: (i) The K_cmgl_-induced type, (ii) the [SC]_*l*_-induced type, and (iii) the [SC]_*l*_ and [SC]_s_-induced type. For the K_cmgl_-induced type, high binding constant for Mg^2+^ reduces the negativity of Ψ_dnl_ (Figure 5G), which increases the concentration of Cl^–^ while decreases the concentration of cations (K^+^ and H^+^) close to the membrane-surface. This causes a larger Cl^–^ flux penetrating the membrane (Supplementary Figure 2B) and a larger concentration of H^+^ kept in the bulk phase (Supplementary Figure 3B). However, a more significant K^+^ flux cannot be notably observed (Supplementary Figure 1B). The K_cmgl_-induced ion flux mediated by the KEA3 is slightly smaller than the controlled (Supplementary Figure 1D). The KEA3 functions to maintain the level of ΔΨ_m_ while decreasing the pH in lumen through the exchange of cations between K^+^ (in) and H^+^ (out) (Kunz et al., 2014; Armbruster et al., 2016; Wang et al., 2017; Wang and Shikanai, 2019). Additionally, the effect of ion flux mediated by the Cl^–^ channel CLCe is trivial (∼10^–4^ mM/s) (Supplementary Figure 2D). This result was also visible in the computing data from Li et al. (2021). Conceivably, more Cl^–^ traveling to the stroma will increase the negativity of Ψ_dns_ (Figure 5D), in addition to the less negative Ψ_dnl_, which ultimately converts the total Donnan potential (ΔΨ_dn_) to negative values (Figure 5A). This, in turn, keeps the steady-level of ΔΨ_m_ only marginally altered by playing an essential counterpart against the highly increased ΔΨ_d_ (Figure 4A) due to the co-function of ΔΨ_h_ (Figure 2A), ΔΨ_k_ (Figure 2D), and ΔΨ_cl_ (Figure 2G). It should also be noted that the kinetics of ΔΨ_dn_, ΔΨ_d_, and ΔΨ_m_ as the light is switched off relaxes in the same pattern as described above. This holds in all simulations for three patterns.

For the [SC]_*l*_-induced type, the consequence is, in fact, antiparallel to those induced by the high binding constant for Mg^2+^, since high abundance of negative charge sites increases the negativity of Ψ_dnl_ (Figure 5H), which in turn increases the concentration of cations (K^+^ and H^+^) while decreasing the concentration of Cl^–^ close to the membrane-surface. This causes a larger transmembrane K^+^ flux (Supplementary Figure 1B) and a smaller concentration of H^+^ stored in the bulk-phase (Supplementary Figure 3B) whereas a significantly reduced Cl^–^ flux cannot be notably observed (Supplementary Figure 2B). This might imply that the Cl^–^ enrichment close to the membrane-surface is likely to be more affected by the “screening” effect caused by cations binding to the surface charges nor the abundance of negative charge sites themselves. Understandably, more K^+^ traveling to the stroma will decreases the negativity of Ψ_dns_ (Figure 5E), in addition to the more negative Ψ_dnl_, which ultimately produce a more positive value for the Donnan potential (ΔΨ_dn_) (Figure 5B). However, under this condition, the co-function of ΔΨ_h_ (Figure 2B), ΔΨ_k_ (Figure 2E), and ΔΨ_cl_ (Figure 2H) makes ΔΨ_d_ a value being enormously negative (Figure 4B), which eventually decreases the steady-level of ΔΨ_m_ (Figure 3B) with an extent a bit larger than in the K_cmgl_-induced type (Figure 3A). This might demonstrate that, by identically enlarging K_cmgl_ or [SC]_*l*_ 10-fold of the controlled, the abundance of the negative charge sites will cause more pronounced effects on regulating ΔΨ_dn_, ΔΨ_d_, and ΔΨ_m_ than the K_cmgl_-induced type.

When concurrently increasing [SC]_*l*_ and [SC]_s_, i.e., for the [SC]_*l*_ and [SC]_s_-induced type, the high abundance of negative charge sites on both surfaces can concomitantly reduce both the negativity of Ψ_dnl_ and Ψ_dns_ (Figures 5F,I). This triggers the kinetic behavior a complexity compared to the K_cmgl_-induced or the [SC]_*l*_-induced type. With the negativity decrease for Ψ_dnl_ and Ψ_dns_, the Cl^–^ flux density is largely reduced (Supplementary Figure 2B) but the enormously increased H^+^ (out)/K^+^ (in) flux conducted by the KEA3 (Supplementary Figure 1D) is observed following a complex dynamic pattern (a sharp rise followed by a deep well till leveling off) as the light is switched on. Intriguingly, the K^+^ flux nearly remains unaffected (Supplementary Figure 1B). The resultant effect of all ion fluxes causes a positive ΔΨ_dn_ with a small amplitude (Figure 5C) and makes the steady-state ΔΨ_d_ reach a value being more positive (Figure 4C). However, it is unparallel to the standard curve but softly decaying till saturating. Similarly, the ΔΨ_m_ bears a resemblance to ΔΨ_d_ but being more positive because it is apparent that the addition of ΔΨ_d_ and ΔΨ_dn_ (being positive) produces the ΔΨ_m_. Taken together, our simulations show that Ψ_d*n*_ can affect the concentration of ions close to the membrane-surface and thus the diffuse potential (ΔΨ_d_) and consequently the total membrane potential (ΔΨ_m_). Therefore, Ψ_dn_ plays a key role on regulating the thylakoid-membrane energization during photosynthesis and should be considered in future studies, which are related to the transthylakoid electrical potential.

### Role of a Mg^2+^ flux through the thylakoid membrane

As noted, thylakoid-harbored non-selective cation pores are not only permeable to K^+^ but also to Mg^2+^ (Enz et al., 1993; Pottosin and Schönknecht, 1996), causing a rise, upon illumination, of Mg^2+^ concentration in the stroma by 1–5 mM (Krause, 1977; Portis, 1981). To check the role of Mg^2+^ on regulating ΔΨ_m_, we additionally merged a Mg^2+^ flux calculated by the GHK equation similar to K^+^ flux into our model and the value of permeability for Mg^2+^ was set at one-third of that for K^+^ based on the measuring data from Pottosin and Schönknecht (1996), i.e., the single channel conductance is 60 pS for K^+^ and 19 pS for Mg^2+^. It turns out that, although several local features of the ΔΨ_m_ (the total membrane potential) trace were altered (data not shown), the general kinetic trend keeps unaffected, which also shows a sharp rise upon illumination before decaying to certain extent of its initial amplitude and then rising again till reaching a steady-state. When the light is switched off, the ΔΨ_m_ curve also shows a sharp fall before returning to the dark baseline. The unaffected kinetic trend might be explained by the fact that more cationic effluxes into stroma would cause a raised dissipation for the diffusion potential difference (ΔΨ_d_). On the other hand, more cations entry into stroma would cause an increase for the negativity of Ψ_dnl_ but a concomitant decrease for the negativity of Ψ_dns_, ultimately triggering a rise of the Donnan potential difference (ΔΨ_dn_), which compensates the loss of ΔΨ_d_. Furthermore, we also conducted the simulations under which principles for regulations by the altered parameters on ΔΨ_m_ remain unchanged as those shown in Figure 3. For instance, the ΔΨ_m_ curve induced by the increased K_cmgl_ (control, ×5, and ×10) was also characterized as a more negative starting point, a higher peak, a faster decay till reaching the steady-state during the light phase as well as a more rapid fall followed by an accelerated relaxation returning to the baseline in the darkness. In essence, the adjustment of ΔΨ_m_ by the Mg^2+^ efflux manifests an “identical” effect as by the K^+^ efflux because they both are cations and merely differ in their valences.

### Donnan potential or surface potential at the thylakoid membrane?

It has been shown that both surfaces of the thylakoid membrane carry fixed negative charges (Barber, 1980b,^1982^). An electrical potential difference is thus established between the membrane surface and the electrolyte solution, which is sufficiently negative to enrich the concentration of cations or to deplete the concentration of anions close to the thylakoid membrane surface. This electric potential has been mathematically computed by two different approaches, i.e., the Donnan equilibrium potential or the Gouy–Chapman double-layer potential (the surface potential is termed as a synonym). As noted above, the surface potential assumes that the thickness of membrane surface charge layer (d_s_) is zero and all membrane fixed charges are only located within the membrane surface. Theoretically, provided d_s_ ≥ 1/κ (κ being the Debye–Hückel parameter), the electric potential in the region far inside the charge layer is practically equal to the Donnan potential (Ohshima and Ohki, 1985; Ohshima and Kondo, 1988b,^1990^). Provided d_s_ ≤ 1/κ, the electric potential at the membrane surface is practically equal to the surface potential. The Donnan potential (d_s_ → ∞) can be converted to the surface potential (d_s_ → 0) undergoing a smooth transition (Ohshima and Kondo, 1990). In essence, the surface potential is a transformer of the Donnan potential as d_s_ is approaching zero (Ohshima and Kondo, 1990). Up to the present, all attempts to estimate d_s_ have been subject to artifacts (Levine et al., 1983). Additionally, using the initial values of our model for all solute species, 1/κ for the thylakoid membrane is calculated as 61.5 nm (see Supplementary Material). Although the characterization of electric potential is dependent on d_s_, it is still adequate to assume the inner space of the chloroplast as a Donnan system because the Gouy–Chapman potential is essentially an alternative “Donnan potential” at the extrema. Additionally, the Donnan potential has particularly been appropriate for studies of ion transport processes through membrane (Ohshima and Ohki, 1985). In our study, at the standard state, the Donnan potential is computed as within ∼−12.5 to ∼−10 mV for the luminal compartment (Ψ_dnl_) (Figure 1C) and ∼−15 to ∼−12.5 mV for the stromal compartment (Ψ_dns_) (Figure 1C), both of which agree with the literature data (Sperelakis, 2012; Qasem et al., 2018).

## Conclusion

By employing an extended photosynthetic model, we investigated the effect of altered cationic binding to the negatively charged thylakoid membrane surface on adjusting the transthylakoid electric potential focusing on a cycle of illumination followed by a dark-adapted period. Consequently, the measured ECS traces were well described by the computing results by assigning the model parameters with the quantitative values taken from the literature. Moreover, the computing data clarified the components of transthylakoid membrane potential, unraveled the functional consequences of altered cationic attachment to the membrane surface on adjusting the transthylakoid electric potential, and revealed the key role played by the Donnan potential in bridging the diffusion potential with the total membrane potential. Ultimately, the compatibility of Donnan theory employed in the chloroplast in conjunction with the interrelationship between the Donnan potential and the Gouy–Chapman potential were discussed. The current model presented in this work can serve as a basis for further extension into a more detailed theoretical model by which multiple variables involved in photosynthesis can be explored.

## Data availability statement

The original contributions presented in this study are included in the article/Supplementary Material, further inquiries can be directed to the corresponding authors.

## Author contributions

HL conceived the study, performed the experiments, constructed the model, performed the simulations, and wrote parts of the manuscript. DL analyzed the experimental data, constructed the model, wrote parts of the manuscript, and supervised the referencing. Both authors contributed to the article and approved the submitted version.

## Supplementary material

Supplementary Figure 1 Simulated responses of the K^+^ flux and the H^+^/K^+^ flux on assigning different values for adjustable parameters. **(A)** 3D view of the K^+^-flux kinetics simulated under control and by 10-fold increasing K_cmgl_, [SC]_*l*_, and [SC]_*l*_ and [SC]_s_, respectively; **(B)** 2D view of the K^+^-flux kinetics simulated under control and by 10-fold increasing K_cmgl_, [SC]_*l*_, and [SC]_*l*_ and [SC]_s_, respectively; **(C)** 3D view of the H^+^/K^+^-flux kinetics simulated under control and by 10-fold increasing K_cmgl_, [SC]_*l*_, and [SC]_*l*_ and [SC]_s_, respectively; **(D)** 2D view of the H^+^/K^+^-flux kinetics simulated under control and by 10-fold increasing K_cmgl_, [SC]_*l*_, and [SC]_*l*_ and [SC]_s_, respectively.

Supplementary Figure 2 Simulated responses of the Cl^–^ flux and the H^+^/Cl^–^ flux on assigning different values for adjustable parameters. **(A)** 3D view of the Cl^–^-flux kinetics simulated under control and by 10-fold increasing K_cmgl_, [SC]_*l*_, and [SC]_*l*_ and [SC]_s_, respectively; **(B)** 2D view of the Cl^–^-flux kinetics simulated under control and by 10-fold increasing K_cmgl_, [SC]_*l*_, and [SC]_*l*_ and [SC]_s_, respectively; **(C)** 3D view of the H^+^/Cl^–^-flux kinetics simulated under control and by 10-fold increasing K_cmgl_, [SC]_*l*_, and [SC]_*l*_ and [SC]_s_, respectively; **(D)** 2D view of the H^+^/Cl^–^-flux kinetics simulated under control and by 10-fold increasing K_cmgl_, [SC]_*l*_, and [SC]_*l*_ and [SC]_s_, respectively.

Supplementary Figure 3 Simulated responses of [H^+^]_*l*_ and [H^+^]_s_ on assigning different values for adjustable parameters. **(A)** 3D view of [H^+^]_*l*_ responses simulated under control and by 10-fold increasing K_cmgl_, [SC]_*l*_, and [SC]_*l*_ and [SC]_s_, respectively; **(B)** 2D view of the [H^+^]_*l*_ responses simulated under control and by 10-fold increasing K_cmgl_, [SC]_*l*_, and [SC]_*l*_ and [SC]_s_, respectively; **(C)** 3D view of the [H^+^]_s_ responses simulated under control and by 10-fold increasing K_cmgl_, [SC]_*l*_, and [SC]_*l*_ and [SC]_s_, respectively; **(D)** 2D view of the [H^+^]_s_ responses simulated under control and by 10-fold increasing K_cmgl_, [SC]_*l*_, and [SC]_*l*_ and [SC]_s_, respectively.

## Abbreviations

ATP: adenosine triphosphate
PMF: proton motive force
NPQ: non-photochemical quenching
PSII: photosystem II
Δ pH: transthylakoid pH difference
ΔΨ_m_: membrane potential difference
ΔΨ_dn_: Donnan potential difference
Ψ_dn_: Donnan potential
Ψ_dnl_: luminal Donnan potential
Ψ_dns_: stromal Donnan potential
ΔΨ_d_: diffusion potential difference
ΔΨ_h_: electric potential difference caused by H^+^ translocation
ΔΨ_k_: electric potential difference caused by K^+^ translocation
ΔΨ_cl_: electric potential difference caused by Cl^–^ translocation
K_cmgl_/K_cmgs_: binding constant of Mg^2+^ attached to negative charge site on luminal/stromal membrane surface
K_pmgl_/K_*pmgs*_: binding constant of Mg^2+^ attached to neutral site on luminal/stromal membrane surface
K_pkl_/K_*pks*_: binding constant of K^+^ attached to neutral site on luminal/stromal membrane surface
K_*phl*_/K_phs_: binding constant of H^+^ attached to neutral site on luminal/stromal membrane-surface
[SC]_*l*_ ([SC]_s_): density of negative charge site on luminal (stromal) membrane surface
[SP]_*l*_ ([SP]_s_): density of neutral site on luminal (stromal) membrane surface
d_s_: thickness of membrane surface charge layer
κ: Debye–Hückel parameter
ECS: electrochromic shift
GHK: Goldman–Hodgkin–Katz
TSRT: transition state rate theory
ρ: density of non-neutralized membrane-fixed negative charge
qE: energy-dependent NPQ of chlorophyll fluorescence
OEC: oxygen evolving complex.
