# Supplementary figures and images for: Analyzing the effect of ion binding to the membrane-surface on regulating the light-induced transthylakoid electric potential (ΔΨm)
Source: Front Plant Sci. 2022 Jul 28;13:945675. doi: 10.3389/fpls.2022.945675 (PMC9366520; doi:10.3389/fpls.2022.945675)

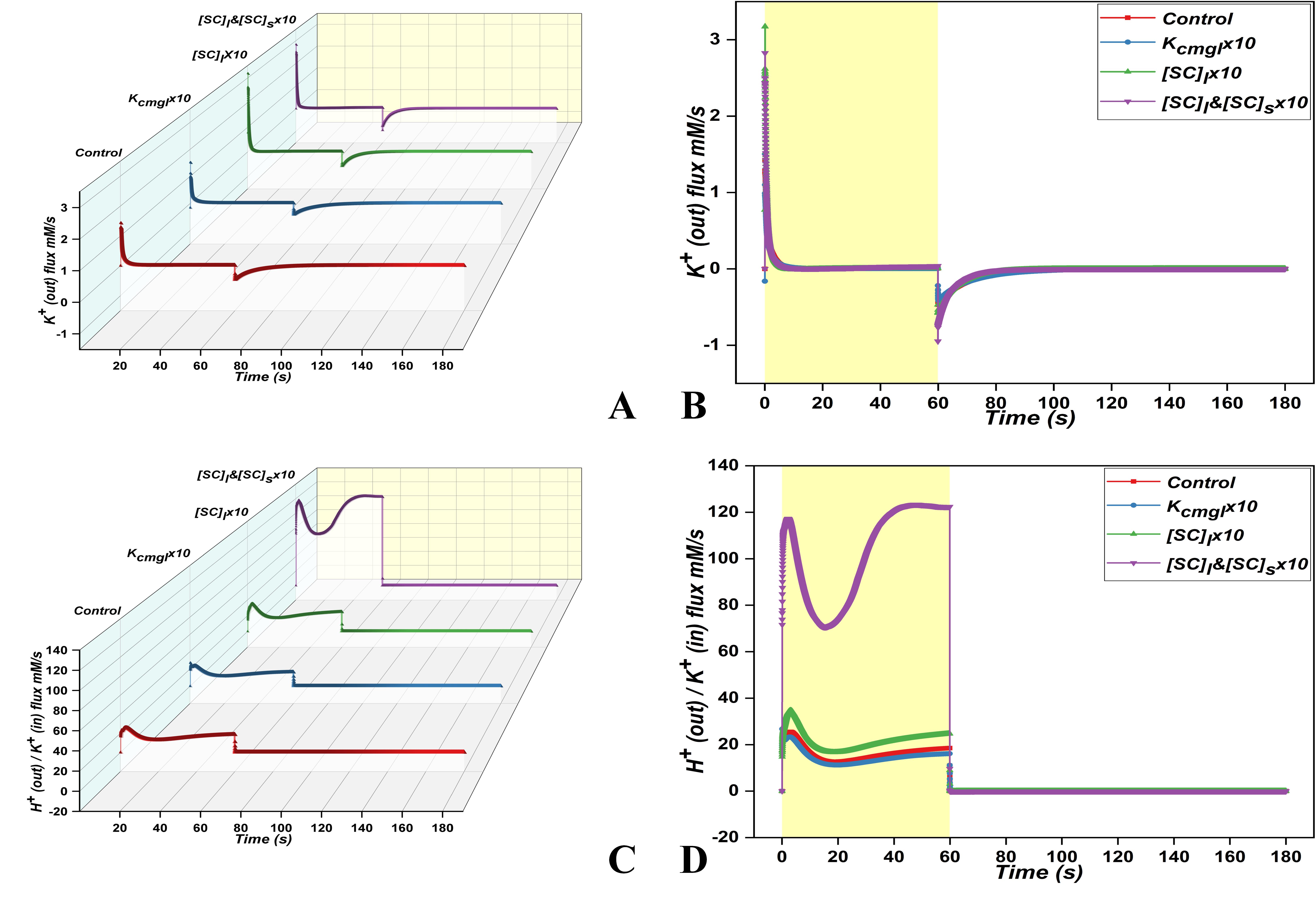

Supplement: Supplementary Figure 1 — Simulated responses of the K+ flux and the H+/K+ flux on assigning different values for adjustable parameters. (A) 3D view of the K+-flux kinetics simulated under control and by 10-fold increasing Kcmgl, [SC]l, and [SC]l and [SC]s, respectively; (B) 2D view of the K+-flux kinetics simulated under control and by 10-fold increasing Kcmgl, [SC]l, and [SC]l and [SC]s, respectively; (C) 3D view of the H+/K+-flux kinetics simulated under control and by 10-fold increasing Kcmgl, [SC]l, and [SC]l and [SC]s, respectively; (D) 2D view of the H+/K+-flux kinetics simulated under control and by 10-fold increasing Kcmgl, [SC]l, and [SC]l and [SC]s, respectively. [file Image_1.JPEG]

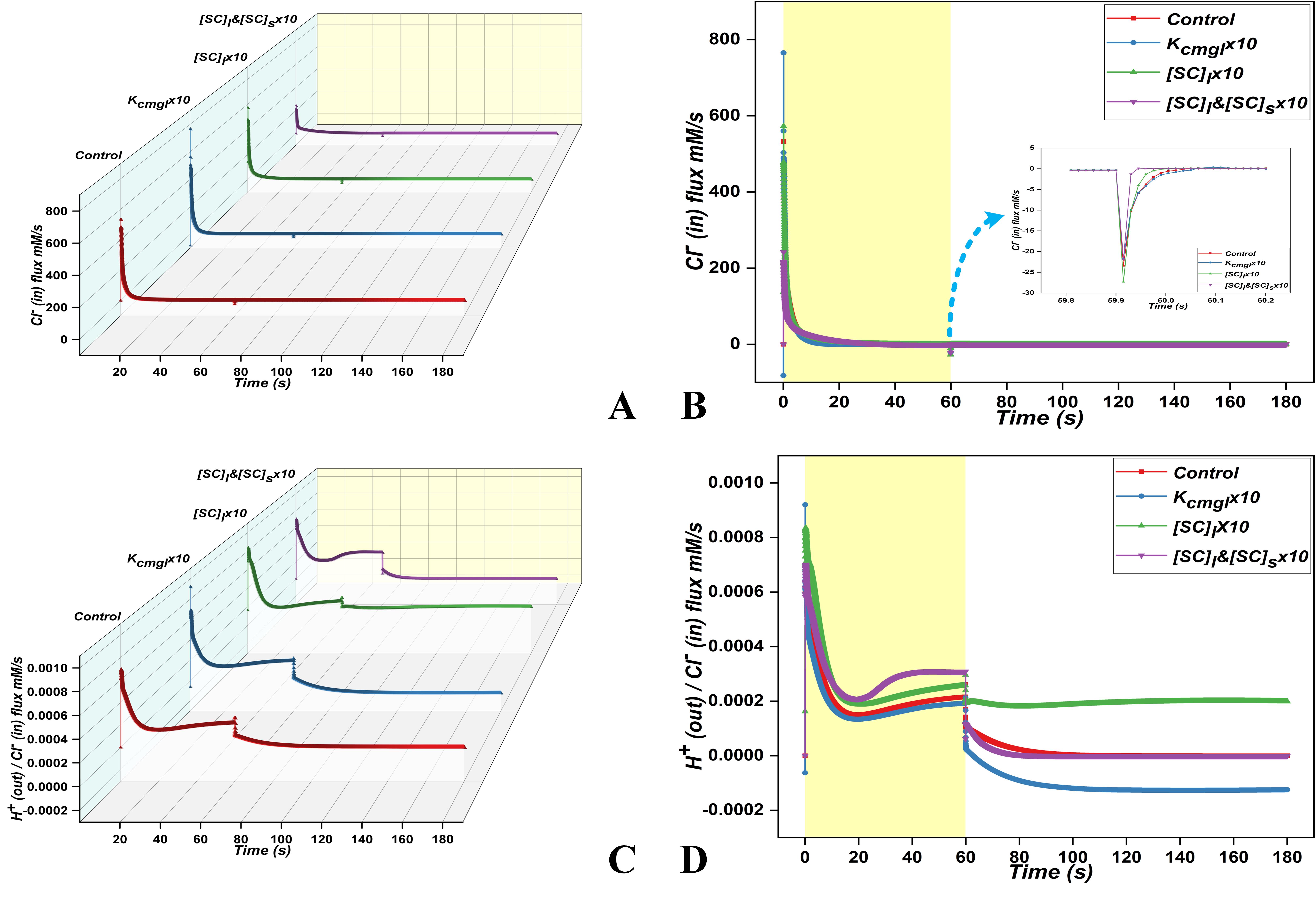

Supplement: Supplementary Figure 2 — Simulated responses of the Cl– flux and the H+/Cl– flux on assigning different values for adjustable parameters. (A) 3D view of the Cl–-flux kinetics simulated under control and by 10-fold increasing Kcmgl, [SC]l, and [SC]l and [SC]s, respectively; (B) 2D view of the Cl–-flux kinetics simulated under control and by 10-fold increasing Kcmgl, [SC]l, and [SC]l and [SC]s, respectively; (C) 3D view of the H+/Cl–-flux kinetics simulated under control and by 10-fold increasing Kcmgl, [SC]l, and [SC]l and [SC]s, respectively; (D) 2D view of the H+/Cl–-flux kinetics simulated under control and by 10-fold increasing Kcmgl, [SC]l, and [SC]l and [SC]s, respectively. [file Image_2.JPEG]

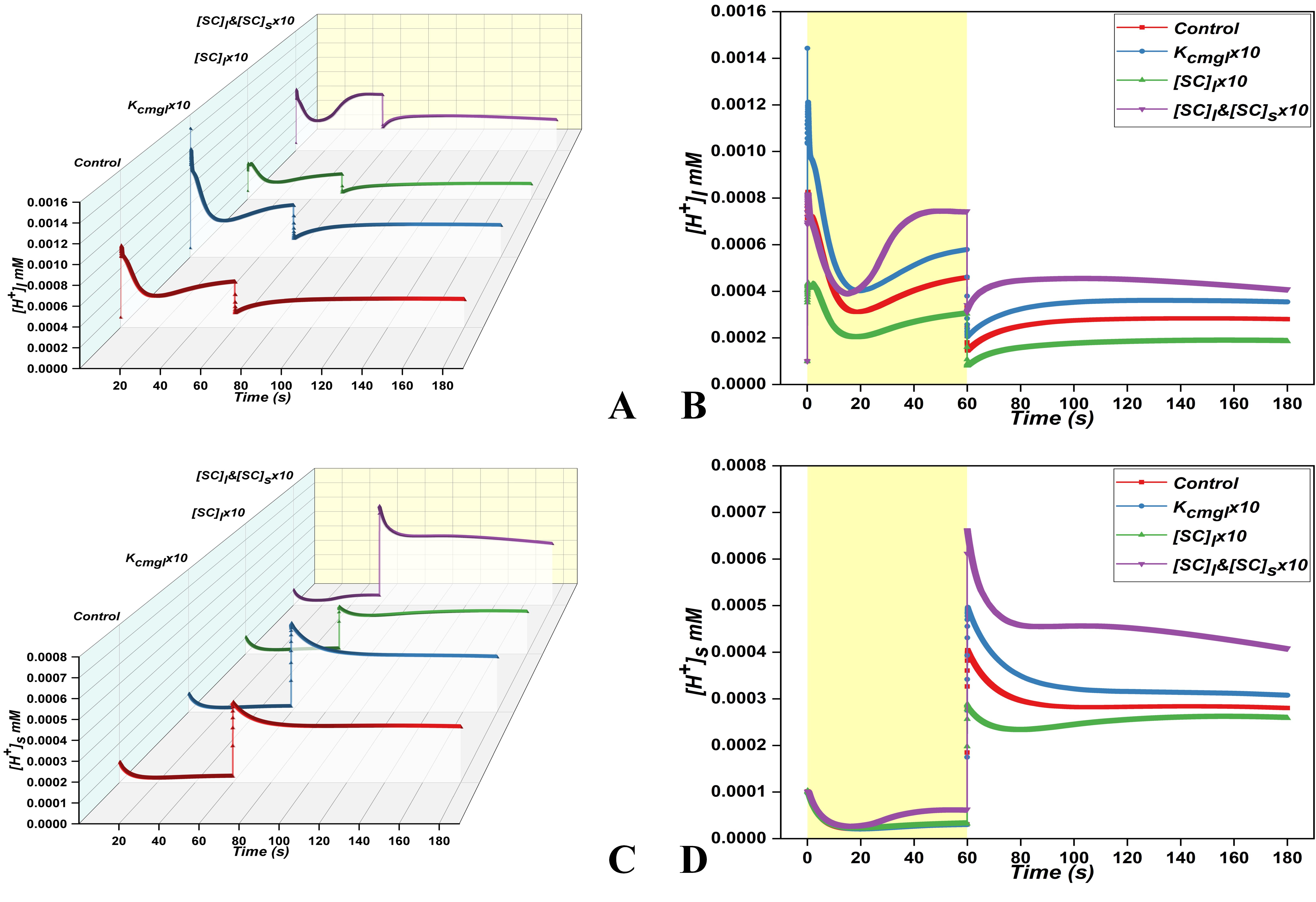

Supplement: Supplementary Figure 3 — Simulated responses of [H+]l and [H+]s on assigning different values for adjustable parameters. (A) 3D view of [H+]l responses simulated under control and by 10-fold increasing Kcmgl, [SC]l, and [SC]l and [SC]s, respectively; (B) 2D view of the [H+]l responses simulated under control and by 10-fold increasing Kcmgl, [SC]l, and [SC]l and [SC]s, respectively; (C) 3D view of the [H+]s responses simulated under control and by 10-fold increasing Kcmgl, [SC]l, and [SC]l and [SC]s, respectively; (D) 2D view of the [H+]s responses simulated under control and by 10-fold increasing Kcmgl, [SC]l, and [SC]l and [SC]s, respectively. [file Image_3.JPEG]
