# Supplementary material for: Analyzing the effect of ion binding to the membrane-surface on regulating the light-induced transthylakoid electric potential (ΔΨm)
Source: Front Plant Sci. 2022 Jul 28;13:945675. doi: 10.3389/fpls.2022.945675 (PMC9366520; doi:10.3389/fpls.2022.945675)
Supplement: Supplementary file 4 [file Data_Sheet_1.pdf]

Analyzing the effect of ion binding to the membrane-surface on regulating the light-induced transthylakoid electric potential ($\Delta\Psi_m$)

Hui Lyu & Dušan Lazár

Supplementary data

Content

Ⅰ. General 2
Ⅱ. Determination of Debye length for the thylakoid membrane 2
Ⅲ. Determination of Donnan potential involving ion attachment to membrane-surface 3
Ⅳ. Electric Potential Difference ($\Delta\Psi_m$) Calculation 5
Ⅴ. Electron transport and proton translocation 5
Ⅵ. Calvin-Benson cycle 11
Ⅶ. Proton efflux pumped by ATP-synthase 18
Ⅷ. Buffer capacity (BC) and pH calculation 19
Ⅸ. Ion fluxes mediated by channels/transporters 20
Ⅹ. Regulatory reactions 21
Ⅺ. Solver 22
Ⅻ. References 23

I. General

The present study is based on our previous models (Lyu and Lazar, 2017a;b) which consists of computational segments of the total electric potential difference across the thylakoid membrane ($\Delta\Psi_m$), electron transport (linear and cyclic electron transport), proton efflux through ATP-synthase, Calvin-Benson cycle, buffer capacity and pH calculation, ion fluxes across the thylakoid membrane and regulatory pathways. In the present work, we introduce the Donnan equilibrium potential to elaborate on the electrical phenomena adjacent to the thylakoid membrane-surface. Overall, Ψ_{dn} can regulate the distribution of ions close to the membrane-surface and thus cause the enrichment of cations and repulsion of anions, consequently adjusting the dynamics of channels/transporters mediated ion fluxes and the proton efflux through ATP-synthase. All the computational components involved in the model are described in the following sections.

II. Determination of Debye length for the thylakoid membrane

It is assumed that the membrane core is covered by a charged layer of thickness d_s. Theoretically, provided $d_s \geq 1/\kappa$ ($1/\kappa$ being the Debye length and κ being the Debye-Hückel parameter), the potential in the region far inside the polyelectrolyte layer is practically equal to the Donnan potential. In short, $1/\kappa$ can be calculated by (Ohshima, 2006):

$$\frac{1}{\kappa} = \frac{1}{\sqrt{\frac{N_A e^2 \sum_{i=1}^{N}(z_i^2 C_i^\infty)}{\varepsilon_r \varepsilon_o kT}}} \qquad S2.1$$

Where N_A is Avogadro number (6.02×10^{23} mol^{-1}), e is the elementary electric charge (1.6×10^{-19} C), ε_r is the relative permittivity of the solution (80), ε_o is the permittivity of a vacuum (8.85×10^{-12} C^2 J^{-1} m^{-1}), k is the Boltzmann constant (1.38×10^{-23} J K^{-1}), T is the absolute temperature (298 K), z_i is the valence of the ith ion (i = 1, 2, …, N), C_i^∞ is the bulk concentration of the ith ion expressed in mM.

The calculation can be specified by loading all kinds of ions (Mg^{2+}, K^+, H^+, Cl^-, OH^-,

B^-) into our model, which is:

$$\frac{1}{\kappa} = \frac{1}{\sqrt{\frac{N_A e^2 [4C_{mg}^{\infty} + C_k^{\infty} + C_h^{\infty} + C_{cl}^{\infty} + C_{oh}^{\infty} + C_b^{\infty}]}{\varepsilon_r \varepsilon_o kT}}} \qquad S2.2$$

The initial concentrations for Mg^{2+}, K^+, H^+, Cl^-, OH^-, and B^- used in our model were taken from the literatures (Van Kooten et al., 1986;Zhu et al., 2013), which are 5 mM, 10 mM, 10^{-4} mM, 1 mM, 10^{-4} mM, and 19 mM, respectively, thus 1/κ for the thylakoid membrane is calculated as 61.5 nm.

III. Determination of Donnan potential involving ion attachment to membrane-surface

The Donnan potential is the electrical potential at which the electroneutrality of the entire system is reached, which can be determined from:

$$\sum_{i=1}^{N} z_i C_i^{\infty} exp\left(-\frac{z_i F \Psi_{dn}}{RT}\right) + \frac{\rho}{F} \times ro = 0 \qquad S3.1$$

Where ρ is the density of the membrane-fixed negative charges. ρ depends in part on intrinsic surface charge density and also includes those solute ions that bind to the membrane-surface. In the present study, the model takes into account 1:1 binding of cations to a negatively charged site (R^- is a representative of [SC] shown in the figures) and a neutral site (P^0 is a representative of [SP] shown in the figures) as described in the following reactions:

$$R^- + I_i^{z_i} = (RI_i)^{z_i - 1} \qquad S3.2.1$$

$$R^- \times I_i^{z_i} \times K_{ci} = (RI_i)^{z_i - 1} \qquad S3.2.2$$

$$P^0 + I_i^{z_i} = (PI_i)^{z_i} \qquad S3.3.1$$

$$P^0 \times I_i^{z_i} \times K_{pi} = (PI_i)^{z_i} \qquad S3.3.2$$

$$I_i^{z_i} = C_i^{\infty} exp\left(-\frac{z_i F \Psi_{dn}}{RT}\right) \qquad S3.4$$

$$\frac{\rho}{F} \times ro = -[R^-] + \sum_{i=1}^{N} (z_i - 1)[(RI_i)^{z_i - 1}] + \sum_{i=1}^{N} z_i [(PI_i)^{z_i}] \qquad S3.5$$

Thus, the calculation can be specified for the Donnan potential combined with the ion binding to the luminal/stromal membrane surface:

$$C_{hl}^{\infty}exp\left(-\frac{F\Psi_{dnl}}{RT}\right)+C_{kl}^{\infty}exp\left(-\frac{F\Psi_{dnl}}{RT}\right)+2C_{mgl}^{\infty}exp\left(-\frac{2F\Psi_{dnl}}{RT}\right)$$
$$-C_{cll}^{\infty}exp\left(\frac{F\Psi_{dnl}}{RT}\right)-C_{ohl}^{\infty}exp\left(\frac{F\Psi_{dnl}}{RT}\right)-C_{bl}^{\infty}exp\left(\frac{F\Psi_{dnl}}{RT}\right)$$
$$-\left(R_l^- - R_l^- \times C_{mgl}^{\infty}exp\left(-\frac{2F\Psi_{dnl}}{RT}\right)\times K_{cmgl}\right.$$
$$-2\times P_l^0\times C_{mgl}^{\infty}exp\left(-\frac{2F\Psi_{dnl}}{RT}\right)\times K_{pmgl}$$
$$\left.-P_l^0\times C_{kl}^{\infty}exp\left(-\frac{F\Psi_{dnl}}{RT}\right)\times K_{pkl}-P_l^0\times C_{hl}^{\infty}exp\left(-\frac{F\Psi_{dnl}}{RT}\right)\times K_{phl}\right)$$
$$=0\qquad S3.6$$

and

$$C_{hs}^{\infty}exp\left(-\frac{F\Psi_{dns}}{RT}\right)+C_{ks}^{\infty}exp\left(-\frac{F\Psi_{dns}}{RT}\right)+2C_{mgs}^{\infty}exp\left(-\frac{2F\Psi_{dns}}{RT}\right)$$
$$-C_{cls}^{\infty}exp\left(\frac{F\Psi_{dns}}{RT}\right)-C_{ohs}^{\infty}exp\left(\frac{F\Psi_{dns}}{RT}\right)-C_{bs}^{\infty}exp\left(\frac{F\Psi_{dns}}{RT}\right)$$
$$-\left(R_s^- - R_s^- \times C_{mgs}^{\infty}exp\left(-\frac{2F\Psi_{dns}}{RT}\right)\times K_{cmgs}\right.$$
$$-2\times P_s^0\times C_{mgs}^{\infty}exp\left(-\frac{2F\Psi_{dns}}{RT}\right)\times K_{pmgs}$$
$$-P_s^0\times C_{ks}^{\infty}exp\left(-\frac{F\Psi_{dns}}{RT}\right)\times K_{pks}$$
$$\left.-P_s^0\times C_{hs}^{\infty}exp\left(-\frac{F\Psi_{dns}}{RT}\right)\times K_{phs}\right)=0\qquad S3.7$$

$\Delta\Psi_{dn}$ is ultimately determined from S3.6 and S3.7, which is:

$$\Delta\Psi_{dn}=\Psi_{dns}-\Psi_{dnl}\qquad S3.8$$

Under the standard condition, R_l^-/R_s^- was assigned the value of 3.63×10^{-5} mol/cm^3 after Barber (1982) (Barber, 1982). P_l^0/P_s^0 was set at 8-fold of R_l^-/R_s^- after Kinraide et al. (1998) (Kinraide et al., 1998). K_{cmgl} and K_{cmgs} were identically set at 30 M^{-1} (Kinraide, 1994;Kinraide et al., 1998). K_{pmgl} and K_{pmgs} were identically set at 30/180 M^{-1}. K_{pkl} and K_{pks} were identically set at 1/180 M^{-1}. K_{phl} and K_{phs} were identically set at 21500/180 M^{-1}. In the present work, binding constants for the neutral site are assumed 1/180 the value for binding to the negative site (Kinraide et al., 1998).

IV. Electric Potential Difference ($\Delta\Psi_m$) Calculation

The numerical calculation of $\Delta\Psi_m$ in and across the membrane is considered as follows:

$$\Delta\Psi_m = \Delta\Psi_d + \Delta\Psi_{dn} \qquad S4.1$$

$$\Delta\Psi_d = \Delta\Psi_h + \Delta\Psi_k + \Delta\Psi_{cl} \qquad S4.2$$

Where, $\Delta\Psi_d$ is the diffusion potential consisting of three components ($\Delta\Psi_h + \Delta\Psi_k + \Delta\Psi_{cl}$) and can be calculated as:

$$\Delta\Psi_d = \Delta\Psi_h + \Delta\Psi_k + \Delta\Psi_{cl} = \frac{RT}{z_h F} ln\left(\frac{[H^+]_i}{[H^+]_o}\right) + \frac{RT}{z_k F} ln\left(\frac{[K^+]_i}{[K^+]_o}\right) + \frac{RT}{z_{cl} F} ln\left(\frac{[Cl^-]_i}{[Cl^-]_o}\right) \qquad S4.3$$

The calculation for $\Delta\Psi_{dn}$ has been introduced in section IV.

V. Electron transport and proton translocation

Electron transport in thylakoid membrane as modeled by Lazár (2009) (Lazár, 2009) and modified for PSII by Lazár and Jablonský (2009) (Lazar and Jablonsky, 2009) was used in this work. Electrons are transported starting from OEC of PSII via the PQ pool, cyt*b*$_6$*f* complex and PC to PSI and further Fd and FNR that reduces $NADP^+$ to NADPH. The detailed scheme for all reactions is shown in Scheme S1.

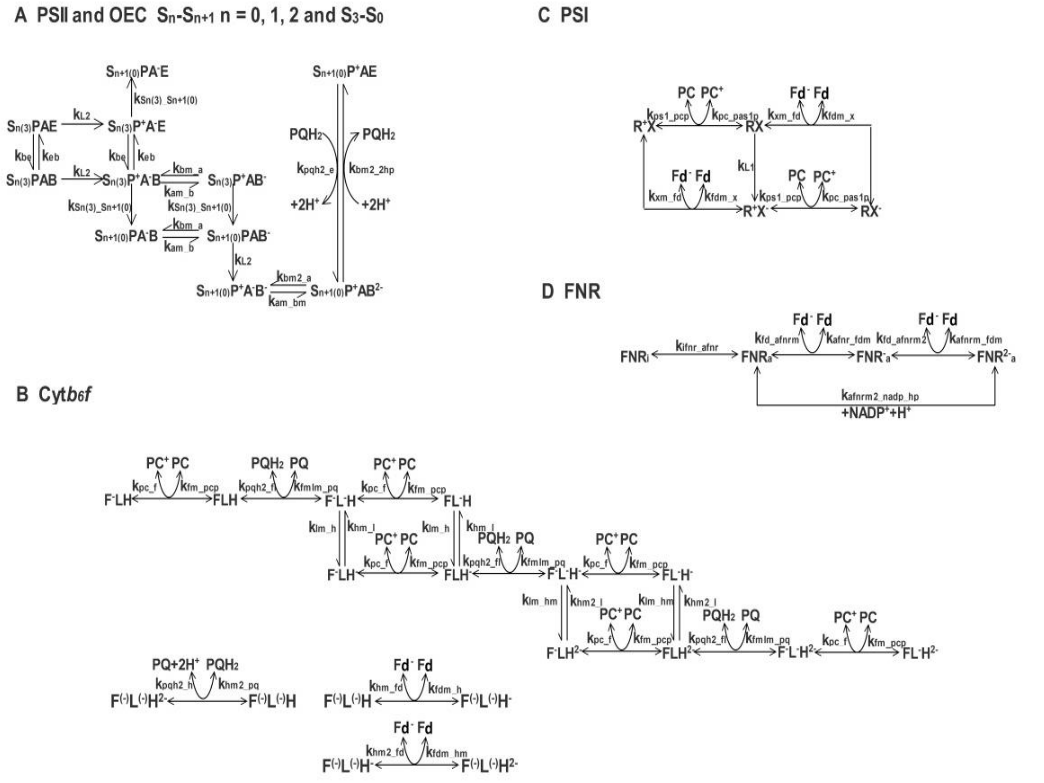

Scheme S1. Details of electron and proton transport reactions occurring in the thylakoid membrane and used in the model. The individual panels (A-D) show the redox reactions related to PSII and OEC (A), cytb_6f (B), PSI (C), and FNR (D). For simplicity, S_n-S_{n+1} (n = 0, 1 and 2) transition of OEC is generalized to avoid the expansion of schematic illustration. This mechanism is suitable also for the S_3-S_0 transition. The reactions describing CET, are shown by three equations representing the various conditions. The abbreviated denotations: S_n – S-states of OEC (n = 0, 1, 2 and 3), P – P680, A – Q_A, B – Q_B, PQH_2 – reduced and protonated PQ, L – b_L heme, H – b_H heme, F – *f* heme, R – P700, X – F_B, FNR_i – inactive FNR, FNR_a – active FNR.

For PSII and adjacent spaces, the S_i-states of OEC, P680, Q_A, Q_B and PQ pool were considered as the redox carriers (Scheme S1-A) connected by following reactions:

1) Light-induced charge separation between P680 and Q_A with rate constants k_{L2} leading to formation of $P680^+$ and Q_A^-. As the time-limiting step of this reaction is rate of excitation formation in P680, k_{L2} reflects this process.
2) Electron donation from $S_{i(3)}$-state (i = 0, 1, 2) of OEC to $P680^+$ leading to formation of $S_{i+1(0)}$-state and P680 with rate constants k_{S0_S1}, k_{S1_S2}, k_{S2_S3}, and k_{S3_S0}. Proton release by OEC in our model, are assumed to takes place during S_0-S_1, S_2-S_3 and S_3-S_0 transitions with the releasing ratio as 1:1:2.

3) Q_B or Q_B^- will be further reduced by Q_A^-. Both reactions are reversible with rate constants of k_{am_b} (forward) and k_{bm_a} (backward), k_{am_bm} (forward) and k_{bm2_a} (backward).

4) The protonation of doubly reduced Q_B (Q_B^{2-}) is described by the second order kinetics as:

$$\text{e.g.,}\quad [S_0PAB^{2-}] + 2[H^+] \rightleftharpoons [S_0PAE] + [PQH_2] \qquad S5.1$$

$$V_{A1} = k_{bm2\_2hp}[S_0PAB^{2-}] - k_{pqh2_e}[S_0PAE]\,\frac{[PQH_2]}{[PQ] + [PQH_2]} \qquad S5.2$$

Where E represents the empty Q_B-pocket in PSII. The rate equation, for simplicity, excludes the two protons but implicitly reflects the stoichiometry of proton combination and liberation. Two protons are combined at this step. Functions related with Cyt*b₆f* and electron carriers (PQ, PC and Fd) in adjacent spaces was described with following reactions (Scheme 1-B):

5) Oxidation of PQH_2 performed by Cytb_6f at lumenal side provides one electron to heme b_L and one electron to heme *f*. This reaction is reversible with forward and backward rate constants k_{pqh2_fl} and k_{fmlm_pq}, respectively. This type of reaction is summarized as:

$$\text{e.g.,}\quad [FLH] + [PQH_2] \rightleftharpoons [F^-L^-H] + [PQ] + 2[H^+] \qquad S5.3$$

$$V_{A3} = k_{pqh2\_fl}[PQH_2][FLH] - k_{fmlm_{pq}}[F^-L^-H]\,\frac{[PQ]}{[PQ] + [PQH_2]} \qquad S5.4$$

Two protons are liberated into lumen by this step.

6) Electron transport from b_L^- to b_H or b_H^- was simulated reversibly with k_{lm_h} or k_{lm_hm} for forward rate constants, k_{hm_l} or k_{hm2_l} for back rate constants.

7) Reduction of PQ by b_H^{2-} of Cytb_6f at stromal side (the Q-cycle) is reversible with forward and backward rate constants k_{hm2_pq} and k_{pqh2_fl}, respectively. This type of reaction is illustrated as:

$$\text{e.g.,}\quad [FLH^{2-}] + [PQ] + 2[H^+] \rightleftharpoons [FLH] + [PQH_2] \qquad S5.5$$

$$V_{A5} = k_{hm2\_pq}[FLH^{2-}]\frac{[PQ]}{[PQ] + [PQH_2]} - k_{pqh2_{fl}}[FLH]\,\frac{[PQH_2]}{[PQ] + [PQH_2]} \qquad S5.6$$

Two protons are combined in this step.

8) Oxidation of reduced heme *f* by PC^+. This reaction is reversible with forward and backward rate constants, k_{fm_pcp} and k_{pc_f}, respectively:

$$\text{e.g.,}\quad [F^{-}LH] + [PC^{+}] \rightleftharpoons [FLH] + [PC] \qquad S5.7$$

$$V_{A7} = k_{fm\_pcp}[F^{-}LH]\frac{[PC^{+}]}{[PC^{+}] + [PC]} - k_{pc_f}[FLH]\frac{[PC]}{[PC] + [PC^{+}]} \qquad S5.8$$

9) Reduction of b_H or b_H^- by Fd^- was described as shown below with forward and backward rate constants, k_{fdm_h} or k_{fdm_hm}, k_{hm_fd} or k_{hm2_fd}, respectively:

$$\text{e.g.}\quad [FLH^{-}] + [Fd^{-}] \rightleftharpoons [FLH^{2-}] + [Fd] \qquad S5.9$$

$$V_{A9} = k_{fdm\_hm}[FLH^{-}]\frac{[Fd^{-}]}{[Fd^{-}] + [Fd]} - k_{hm2_{fd}}[FLH^{2-}]\frac{[Fd]}{[Fd^{-}] + [Fd]} \qquad S5.10$$

Reactions related to PSI and FNR are shown in Scheme S1-C and S1-D, respectively, and are specified as follows:

10) Light-induced charge separation in PSI with rate constants kL1 leading to formation of $P700^+$ and F_B^-.

11) $P700^+$ is reduced by PC. The forward and backward rate constants are k_{pc_ps1p} and k_{ps1_pcp}, respectively. This type of reaction is summarized as:

$$\text{e.g.,}\quad [R^{+}X] + [PC] \rightleftharpoons [RX] + [PC^{+}] \qquad S5.11$$

$$V_{A11} = k_{pc\_ps1p}[R^{+}X]\frac{[PC]}{[PC^{+}] + [PC]} - k_{ps1_{pcp}}[RX]\frac{[PC^{+}]}{[PC] + [PC^{+}]} \qquad S5.12$$

12) F_B^- of PSI is oxidized by Fd. The rate constants for forward and backward reactions are k_{xm_fd} and k_{fdm_x}, respectively. This type of reaction can be expressed as:

$$\text{e.g.}\quad [RX^{-}] + [F_D] \rightleftharpoons [RX] + [F_D^{-}] \qquad S5.13$$

$$V_{A13} = k_{xm\_fd}[RX^{-}]\frac{[F_D]}{[F_D^{-}] + [F_D]} - k_{fdm_x}[RX]\frac{[F_D^{-}]}{[F_D^{-}] + [F_D]} \qquad S5.14$$

Reactions connected with FNR in the model were are as follows:

13) Activation of inactive FNR with rate constant kifnr_afnr.

14) Reversible electron transport from Fd^- to FNR_a or FNR_a^- is simulated with the forward and backward rate constants: k_{afnr_fdm} or k_{fd_afnrm}, k_{afnrm_fdm} or k_{fd_afnrm2}, respectively. This type of reaction is expressed as:

$$\text{e.g.,}\quad [FNR_a^{-}] + [Fd^{-}] \rightleftharpoons [FNR_a^{2-}] + [Fd] \qquad S5.15$$

$$V_{A15} = k_{afnrm\_fdm}[FNR_a^{-}]\frac{[Fd^{-}]}{[Fd^{-}] + [Fd]} - k_{fd_{afnrm}}[FNR_a^{2-}]\frac{[Fd]}{[Fd^{-}] + [Fd]} \qquad S5.16$$

15) Oxidation of FNR_a^{2-} to FNR is described with rate constant $k_{afnrm2\_nadp\_hp}$. This reaction leads to the formation of NADPH from $NADP^+$, combing one proton,

which can be described as:

$$[FNR_a^{2-}] + [NADP^+] + [H^+] \longrightarrow [FNR_a] + [NADPH] \qquad S5.17$$

$$V_{A17} = k_{afnrm2\_nadp\_hp}[FNR_a^{2-}]\frac{[NADP^+]}{[NADP^+] + [NADPH]} \qquad S5.18$$

The proton fluxes involved in calculations of pH_i and pH_o are as follows:

$$J_{OEC} = k_{s0\_s1}\left[\sum S_0 P^+\right] + k_{s2_{s3}}\left[\sum S_2 P^+\right] + 2k_{s3_{s0}}\left[\sum S_3 P^+\right] \qquad S5.19$$

$$J_{PQH_2} = 2k_{pqh2\_fl}\frac{[PQH_2]}{[PQ] + [PQH_2]}\left[\sum FL\right] - 2k_{fmlm_{pq}}\frac{[PQ]}{[PQ] + [PQH_2]}\left[\sum F^-L^-\right] \qquad S5.20$$

$$J_{Q_B^{2-}} = -2k_{bm2_{2hp}}\left[\sum B^{2-}\right] + 2k_{pqh2_e}\frac{[PQH_2]}{[PQ] + [PQH_2]}\left[\sum E\right] \qquad S5.21$$

$$J_{Q_{cycle}} = -2k_{hm2\_pq}\frac{[PQ]}{[PQ] + [PQH_2]}\left[\sum H^{2-}\right] + 2k_{pqh2\_h}\frac{[PQH_2]}{[PQ] + [PQH_2]}\left[\sum H\right] \qquad S5.22$$

$$J_{NADPH} = k_{fnram2\_nadp\_hp}[FNR_a^{2-}]\frac{[NADP^+]}{[NADP^+] + [NADPH]} \qquad S5.23$$

$$J_{CBC} = k_{3f}[BPGA][NADPH] - k_{3b}[NADP^+][GAP][Pi] \qquad S5.24$$

Initial concentration of the variables and values of the rate constants

Absolute values for variables were adopted replacing the relative ratio assigned for the initial concentration shown in Lazár (2009). The initial conditions (for a dark-adapted state) for the simulation were assumed to be: OEC being in S_1-state, Q_A and Q_B completely oxidized, P680 fully reduced, half PQ pool reduced and half oxidized, hemes b_L, b_H and f fully oxidized, PC and P700 fully reduced, F_B and Fd fully oxidized, and FNR fully inactive. The stoichiometric ratio for individual pigment-protein complexes and particular electron carriers used in the model are as follows:

$$[S_1PAB]:[PQ]:[PQH_2]:[FLH]:[PC]:[RX]:[Fd]:[FNR_i]$$
$$= 1:3:3:1:3:0.625:3:3 \qquad S5.25$$

Moreover, initial [S_1PAB] was estimated to be 9.26×10^{-3} mM. Thus, the initial variables were obtained. The rate constants of the electron transport were taken from Lazár (2009) (Lazár, 2009) and are shown in Table S1.

Table S1. Rate constants of the electron transport.

Rate Constant	Value (s^{-1})
k_{L2}	1500
k_{s0_s1}	20000
k_{s1_s2}	10000
k_{s2_s3}	3300
k_{s3_s0}	1000
k_{be}	100
k_{eb}	100
k_{am_b}	3500
k_{bm_a}	175
k_{am_bm}	1750
k_{bm2_a}	35
k_{bm2_2hp}	500
k_{pqh2_e}	100
k_{pqh2_fl}	500
k_{fmlm_pq}	100
k_{lm_h}	1000
k_{hm_l}	10
k_{lm_hm}	1000
k_{hm2_l}	10
k_{hm2_pq}	100
k_{pqh2_h}	10
k_{fm_pcp}	100
k_{pc_f}	10
k_{pc_ps1p}	100
k_{ps1_pcp}	10
k_{L1}	750
k_{xm_fd}	100

k_{fdm_x}	10
k_{fdm_h}	100
k_{hm_fd}	10
k_{fdm_hm}	100
k_{hm2_fd}	10
k_{ifnr_afnr}	10
k_{afnr_fdm}	50
k_{fd_afnrm}	5
k_{afnrm_fdm}	50
k_{fd_afnrm2}	5
$k_{afnr\_nadp\_hp}$	220

VI. Calvin-Benson cycle

Calvin-Benson cycle (CBC) was computed exploiting the models modified by Poolman et al. (2000, 2004) (Poolman et al., 2000;Poolman et al., 2004) where the model configuration was originally developed by Pettersson and Ryde-Pettersson (1988) (Pettersson and Ryde-Pettersson, 1988), afterwards upgraded by Pettersson (1997) (Pettersson, 1997) by integration of the photorespiratory glycolate pathway. The detailed scheme for CBC used in our work is shown in Scheme S2. All the equations involved in CBC were as follows:

$S6.1\ \ RUBP + CO_2(O_2) \longrightarrow 2PGA(PGA)$

In order to reflect the relationship of CBC turnover with pH_o alteration in simulations, the following equation was suggested in the calculation of CO_2 carboxylation.

$$f_{CBC} = \frac{e^{(pH_o-7)} - 1}{e^{0.8} - 1} \qquad S6.2$$

$$E1_{co2} = f_{CBC} \times k_{11\_rubp} \times \frac{[RUBP]}{\left([RUBP] + k_{m11}(1 + [PGA]/k_{i11} + [FBP]/k_{i12} + [SBP]/k_{i13} + [Pi]/k_{i14} + [NADPH]/k_{i15})\right)} \times \left(([CO_2]/k_{m12\_co2})/(1 + [CO_2]/k_{m12\_co2} + [O_2]/k_{m13\_o2})\right) \qquad S6.3$$

$$E1_{o2} = E1_{co2}([O_2]/(k_{sc}[CO_2])) \quad S6.4$$

$$E1_{net\_co2} = E1_{co2} - 0.5 \times E1_{o2} - k_{12_{dr}} \quad S6.5$$

$E1_{net\_co2}$ represents the net CO_2 uptake rate, k_{12_dr} denotes the day respiration rate.

$$S6.6\ \ PGA + ATP \rightleftharpoons BPGA + ADP$$

$$E2 = k_{2f}[PGA][ATP] - k_{2b}[BPGA][ADP] \quad S6.7$$

$$S6.8\ \ BPGA + NADPH + H^+ \rightleftharpoons GAP + NADP^+ + Pi$$

$$E3 = k_{3f}[BPGA][NADPH] - k_{3b}[GAP][NADP^+][Pi] \quad S6.9$$

$$S6.10\ \ GAP \rightleftharpoons DHAP$$

$$E4 = k_{4f}[GAP] - k_{4b}[DHAP] \quad S6.11$$

$$S6.12\ GAP + DHAP \rightleftharpoons FBP$$

$$E5 = k_{5f}[GAP][DHAP] - k_{5b}[FBP] \quad S6.13$$

$$S6.14\ FBP \longrightarrow F6P + Pi$$

$$E6 = \frac{k_{61}[FBP]}{\left([FBP] + k_{m61}(1 + [F6P]/k_{i61} + [Pi]/k_{i62})\right)} \quad S6.15$$

$$S6.16\ \ GAP + F6P \rightleftharpoons X5P + E4P$$

$$E7 = k_{7f}[GAP][F6P] - k_{7b}[X5P][E4P] \quad S6.17$$

$$S6.18\ \ DHAP + E4P \rightleftharpoons SBP$$

$$E8 = k_{8f}[DHAP][E4P] - k_{8b}[SBP] \quad S6.19$$

$$S6.20\ \ SBP \longrightarrow Pi + S7P$$

$$E9 = \frac{k_{91}[SBP]}{\left([SBP] + k_{m91}(1 + [Pi]/k_{i91})\right)} \quad S6.21$$

$$S6.22\ \ S7P + GAP \rightleftharpoons R5P + X5P$$

$$E10 = k_{10f}[S7P][GAP] - k_{10b}[R5P][X5P] \quad S6.23$$

$$S6.24\ \ R5P \rightleftharpoons RU5P$$

$$E11 = k_{11f}[R5P] - k_{11b}[RU5P] \quad S6.25$$

$$S6.26\ \ X5P \rightleftharpoons RU5P$$

$$E12 = k_{12f}[X5P] - k_{12b}[RU5P] \quad S6.27$$

$$S6.28\ \ RU5P + ATP \longrightarrow RUBP + ADP$$

$$E13 = \frac{k_{131}[RU5P][ATP]}{\left([RU5P] + k_{m131}\left(1 + \frac{[PGA]}{k_{i131}} + \frac{[RUBP]}{k_{i132}} + \frac{[Pi]}{k_{i133}}\right)\right)\left([ATP]\left(1 + \frac{[ADP]}{k_{i134}}\right) + k_{m132}\left(1 + \frac{[ADP]}{k_{i135}}\right)\right)} \quad S6.29$$

$S6.30\ \ F6P \rightleftharpoons G6P$

$$\mathrm{E}14 = k_{14f}[F6P] - k_{14b}[G6P] \quad S6.31$$

$S6.32\ \ G6P \rightleftharpoons G1P$

$$\mathrm{E}14 = k_{15f}[G6P] - k_{15b}[G1P] \quad S6.33$$

$S6.34\ \ ATP + G1P \longrightarrow Starch + ADP + 2Pi$

$$E16 = \frac{k_{161}[G1P][ATP]}{([G1P] + k_{m161})\left(\left(1 + \frac{[ADP]}{k_{i161}}\right)([ATP] + k_{m162}) + \frac{k_{m162}[Pi]}{k_{i162}[PGA] + k_{i163}[F6P] + k_{i164}[FBP]}\right)} \quad S6.35$$

$S6.36\ \ Starch + Pi \longrightarrow G1P$

$$E17 = \frac{k_{171}[Pi]}{[Pi] + k_{m171}\left(1 + \frac{[G1P]}{k_{i171}}\right)} \quad S6.37$$

$S6.38\ \ DHAP + Pi_{cy} \longrightarrow DHAP_{cy} + Pi$

$S6.39\ \ GAP + Pi_{cy} \longrightarrow GAP_{cy} + Pi$

$S6.40\ \ PGA + Pi_{cy} \longrightarrow PGA_{cy} + Pi$

$$F_{trans} = 1 + \left(1 + \frac{k_{trans\_pi\_cy}}{[Pi_{cy}]}\right)\left(\frac{[Pi]}{k_{trans\_pi}} + \frac{[PGA]}{k_{trans\_pga}} + \frac{[GAP]}{k_{trans\_gap}} + \frac{[DHAP]}{k_{trans\_dhap}}\right) \quad S6.41$$

$$E18 = \frac{k_{181}[DHAP]}{F_{trans}k_{trans\_dhap}} \quad S6.42$$

$$E19 = \frac{k_{201}[GAP]}{F_{trans}k_{trans\_gap}} \quad S6.43$$

$$E20 = \frac{k_{191}[PGA]}{F_{trans}k_{trans\_pga}} \quad S6.44$$

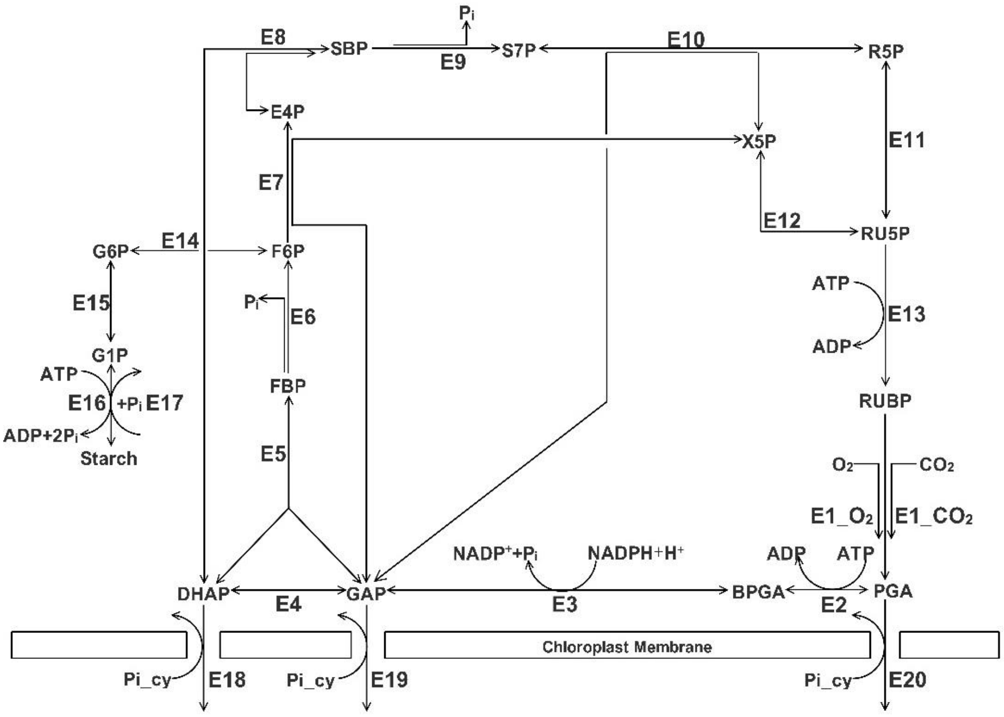

Scheme S2. Details of Calvin-Benson cycle reactions occurring in the stroma and used in the model. CO_2 and external free phosphate have fixed concentrations. The abbreviations: BPGA− 1,3-bis-phosphoglycerate, DHAP− dihydroxyacetone-3-phosphate, E4P− erythrose-4-phosphate, F6P− fructose-1,6-*bis*phosphate, G1P− glucose-1-phosphate, G6P− glucose-6-phosphate, GAP− glyceraldehyde-3-phosphate, R5P− ribose-5-phosphate, Ru5P− ribose-5-phosphate, RuBP− ribulose-1,5-bisphosphate, 2-PGA− 2-phosphoglycerate, PGA− 3-phosphoglycerate, S7P− sedoheptulose-7-phosphate, SBP− sedoheptulose-1,7-bisphosphate, X5P− xylulose 5-phosphate.

The kinetic parameters of the CBC and initial concentrations of the CBC variables

Table S2 and Table S3 show quantitative values of the kinetic parameters in the CBC and initial concentrations of the CBC variables, respectively.

Table S2. Quantitative Values for parameters involved in the CBC.

Parameter	Value (Unit)	Source
k_{11_rubp}	324 umol h^{-1} $(mgchl)^{-1}$	a
k_{m11}	0.02 mM	a
k_{i11}	0.84 mM	a

k_{i12}	0.04 mM	a
k_{i13}	0.075 mM	a
k_{i14}	0.9 mM	a
k_{i15}	0.07 mM	a
k_{m12_co2}	270 ubar	b
k_{m13_o2}	400 mbar	b
k_{sc}	2360	b
k_{12_dr}	3 umol h^{-1} $(mgchl)^{-1}$	b
k_{2f}	1.3×10^{8} $(mM)^{-1}$ s^{-1}	c
k_{2b}	2.5×10^{9} $(mM)^{-1}$ s^{-1}	c
k_{3f}	2.1×10^{9} $(mM)^{-1}$ s^{-1}	c
k_{3b}	4×10^{10} $(mM)^{-2}$ s^{-1}	c
k_{4f}	102 s^{-1}	c
k_{4b}	3.3 s^{-1}	c
k_{5f}	3.37×10^{11} $(mM)^{-1}$ s^{-1}	c
k_{5b}	25 s^{-1}	c
k_{61}	200 umol h^{-1} $(mgchl)^{-1}$	a
k_{m61}	0.03 mM	a
k_{i61}	0.7 mM	a
k_{i62}	12 mM	a
k_{7f}	1.01×10^{11} $(mM)^{-1}$ s^{-1}	c
k_{7b}	6.25×10^{11} $(mM)^{-1}$ s^{-1}	c
k_{8f}	8.4×10^{9} $(mM)^{-1}$ s^{-1}	c
k_{8b}	0.5 s^{-1}	c
k_{91}	40 umol h^{-1} $(mgchl)^{-1}$	a
k_{m91}	0.013 mM	a
k_{i91}	12 mM	a
k_{10f}	2×10^{13} $(mM)^{-1}$ s^{-1}	c
k_{10b}	5×10^{13} $(mM)^{-1}$ s^{-1}	c

k_{11f}	200.6 s^{-1}	c
k_{11b}	200 s^{-1}	c
k_{12f}	252 s^{-1}	c
k_{12b}	200 s^{-1}	c
k_{131}	1000 umol h^{-1} $(mgchl)^{-1}$	a
k_{m131}	0.05 mM	a
k_{m132}	0.05 mM	a
k_{i131}	2 mM	a
k_{i132}	0.7 mM	a
k_{i133}	4 mM	a
k_{i134}	2.5 mM	a
k_{i135}	4 mM	a
k_{14f}	1.002 s^{-1}	c
k_{14b}	0.33 s^{-1}	c
k_{15f}	0.335 s^{-1}	c
k_{15b}	5 s^{-1}	c
k_{161}	40 umol h^{-1} $(mgchl)^{-1}$	a
k_{m161}	0.08 mM	a
k_{m162}	0.08 mM	a
k_{i161}	10 mM	a
k_{i162}	0.1 mM	a
k_{i163}	0.02 mM	a
k_{i164}	0.02 mM	a
k_{171}	40 umol h^{-1} $(mgchl)^{-1}$	a
k_{m171}	0.1 mM	a
k_{i171}	0.05 mM	a
$k_{trans\_pi\_cy}$	0.75 mM	a
k_{trans_pi}	0.63 mM	a
k_{trans_pga}	0.25 mM	a

k_{trans_gap}	0.075 mM	a
k_{trans_dhap}	0.077 mM	a
k_{181}	250 umol h^{-1} $(mgchl)^{-1}$	a
k_{191}	250 umol h^{-1} $(mgchl)^{-1}$	a
k_{201}	250 umol h^{-1} $(mgchl)^{-1}$	a

Table S3. Initial concentrations for the CBC variables.

Parameter	Value (Unit)	Source
RUBP	0.5 mM	C
PGA	4.5 mM	c
BPGA	0.15 mM	c
GAP	0.01 mM	c
DHAP	0.3 mM	c
FBP	0.03 mM	c
F6P	1.5 mM	c
E4P	0.4 mM	c
X5P	0.004 mM	c
SBP	1.6 mM	c
S7P	0.005 mM	c
R5P	0.006 mM	c
RU5P	0.002 mM	c
G6P	3 mM	c
G1P	0.2 mM	c
Starch	1 mM	c
$NADP^+$	0.79 mM	a
NADPH	0.42 mM	e
Pi	0.9 mM	d
ADP	0.82 mM	d
ATP	0.68 mM	d

CO_2	300 μbar	b
O_2	210 mbar	b
Pi_cy	0.5 mM	a

a-Pettersson and Ryde-Pettersson, 1988 (Pettersson and Ryde-Pettersson, 1988); b-Pettersson, 1997 (Pettersson, 1997); c-Poolman et al. 2000, 2004 (Poolman et al., 2000;Poolman et al., 2004); d-Igamberdiev et al. 2001 (Igamberdiev and Kleczkowski, 2001); e-Giersch et al. 1980 (Giersch et al., 1980)

VII. Proton efflux pumped by ATP-synthase

The mechanism for proton efflux pumped by ATP-synthase was modeled according to the equations previously proposed by Kocks and Ross (1995) (Kocks and Ross, 1995):

$$J_{atp} = C_{atp}\frac{\left(S_{atp}\Delta\tilde{\mu}_{H^+} - \Delta G_{atp}\right)}{RT}O_{atp} \qquad S7.1$$

$$\Delta\tilde{\mu}_{H^+} = \Delta\Psi F + 2.3RT\Delta pH \qquad S7.2$$

$$O_{atp} = \frac{1}{1 + e^{\left(14 + S_{atp}\frac{(-\Delta\tilde{\mu}_{H^+})}{RT}\right)}} \qquad S7.3$$

Where C_{atp} represents the conductance of ATP-synthase (mol cm^{-2} s^{-1}), S_{atp} is the stoichiometric ratio of H^+/ATP and ratio of 14/3 determined by atomic force microscopy (Müller et al. 2001) (Muller et al., 2001) was used in the calculation, $\Delta\tilde{u}_{H+}$ denotes the electrochemical potential (J mol^{-1}), ΔG_{atp} represents the energy barrier for ATP synthesis (J mol^{-1}) which is assigned the value of 40000 J mol^{-1}. O_{atp} represents Boltzmann distribution of ATP-synthase opening (0~1) derived from the experimental fitting data (Kocks and Ross, 1995) (Kocks and Ross, 1995). R and T have their usual meanings.

VIII. Buffer capacity (BC) and pH calculation

The calculation for buffer capacities

Formulae used to calculate buffer capacitates are developed below:

$$[H^+]_i + [B^-]_i^s \rightleftharpoons [HB]_i^s \qquad S8.1$$

$$K_i^s = \frac{[H^+]_i[B^-]_i^s}{[HB]_i^s} \qquad S8.2$$

$$[B]_i^s = [B^-]_i^s + [HB]_i^s \qquad S8.3$$

$$[HB]_i^s = \frac{[B]_i^s[H^+]_i}{K_i^s + [H^+]_i} \qquad S8.4$$

Since the definition of BC_i (mM pH^{-1}) is given by:

$$BC_i = \frac{d([H^+]_i + \sum_{s=1}^{n}[HB]_i^s)}{d(pH)_i} \qquad S8.5$$

Thus, BC_i may be solved as:

$$BC_i = \frac{d\left([H^+]_i + \frac{[B]_i[H^+]_i}{K_i + [H^+]_i}\right)}{d(pH)_i} \qquad S8.6$$

Similarly, BC_o can be obtained:

$$BC_o = \frac{d\left([H^+]_o + \frac{[B]_o[H^+]_o}{K_o + [H^+]_o}\right)}{d(pH)_o} \qquad S8.7$$

The subscript '*i*' represents the inner space. The superscript '*s*' represents buffer species (here, s = 1). $[B]$ symbolizes overall concentration of buffer groups and K symbolizes averaged dissociation constant of buffer species. The experimental results (Heldt et al. 1973) proved $K_i = 5.5$ for lumen and $K_O = 6.8$ for stroma, and it reveals that the buffer concentration $[B]_i$ is around four times higher than $[B]_o$ explored in the same work. $[B]_i$ was once assumed as 300 mM (Van Kooten et al., 1986).

The calculation for the intracellular and extracellular pH

The pH is calculated after buffering the sum of the proton fluxes that decrease or increase the accumulation of mobile protons in both reservoirs. This gives:

$$pH_i = \frac{-1}{BC_i}\left(J_{OEC} + J_{PQH_2} - J_{ATP} - J_{leak}^{ls}\right) \qquad S8.8$$

$$pH_o = \frac{-1}{BC_o}\left(J_{ATP} + J_{leak}^{ls} - J_{Q_B^{2-}} - J_{Q_{cycle}} - J_{NADPH} - J_{CBC} - J_{leak}^{sc}\right) \qquad S8.9$$

Proton 'slip' across membrane into stroma was simply calculated as a difference of proton diffusion potential between lumen and stroma, which can be expressed as:

$$J_{leak}^{ls} = k_{leak}^{ls}([H^+]_i - [H^+]_o) \qquad S8.10$$

Where k_{leak}^{ls} means rate constant of proton leakage being best estimated as 10 s^{-1}. $[H^+]_i$ and $[H^+]_o$ symbolize proton concentrations in lumen and stroma, respectively. Similarly, the proton leakage from stroma to cytoplasm was calculated in the same way which leads to:

$$J_{leak}^{sc} = k_{leak}^{sc}([H^+]_o - [H^+]_i) \qquad S8.11$$

Where k_{leak}^{sc} is assigned the value of 200 s^{-1}.

IX. Ion fluxes mediated by channels/transporters

For the ion flux mediated by voltage-dependent K^+ channel V-K^+ and voltage-dependent channel VCCN1, the modified Goldman–Hodgkin–Katz (GHK) flux equation from Van Kooten and co-workers (Van Kooten et al., 1986), intermingled with the effect of Ψ_{dn}-induced cation enrichment or anion repulsion, was combined here to compute the ion fluxes:

For voltage-dependent K^+ channel V-K^+:

$$J_k = P_{k\_1} \times C_{kl}^{\infty} \times exp^{\frac{-F\Psi_{dnl}}{RT}} \times exp^{\frac{F\Delta\Psi_m}{RT}} - P_{k_2} \times C_{ks}^{\infty} \times exp^{\frac{-F\Psi_{dns}}{RT}} \times exp^{\frac{-F\Delta\Psi_m}{RT}} \qquad S9.1$$

For voltage-dependent Cl^- channel VCCN1:

$$J_{cl} = P_{cl\_1} \times C_{cll}^{\infty} \times exp^{\frac{F\Psi_{dnl}}{RT}} \times exp^{\frac{F\Delta\Psi_m}{RT}} - P_{cl_2} \times C_{cls}^{\infty} \times exp^{\frac{F\Psi_{dns}}{RT}} \times exp^{\frac{-F\Delta\Psi_m}{RT}} \qquad S9.2$$

For the H^+/K^+ antiporter KEA3, the equation proposed by Sukhov et al. (2009, 2013, 2015) (Sukhov and Vodeneev, 2009;Sukhov et al., 2013;Sukhov et al., 2015) merged with the effect of Ψ_{dn}-induced cation enrichment or anion repulsion was integrated into the whole model:

$$J_{h/k} = P_{h/k\_1} \times C_{hl}^{\infty} \times exp^{\frac{-F\Psi_{dnl}}{RT}} \times C_{ks}^{\infty} \times exp^{\frac{-F\Psi_{dns}}{RT}} - P_{\frac{h}{k_2}} \times C_{hs}^{\infty} \times exp^{\frac{-F\Psi_{dns}}{RT}} \times C_{kl}^{\infty} \times exp^{\frac{-F\Psi_{dnl}}{RT}} \qquad S9.3$$

For the Cl^- channel CLCe, its function is possibly related to the Cl^- homeostasis across the thylakoid membrane (Li et al., 2021). The mechanism of CLCe was recently modeled by Li and co-workers (Li et al., 2021) and modified in our model:

$$J_{h/cl} = P_{h/cl\_1} \times C_{hl}^{\infty} \times exp^{\frac{-F\Psi_{dnl}}{RT}} \times C_{cls}^{\infty} \times exp^{\frac{F\Psi_{dns}}{RT}} - P_{\frac{h}{cl_2}} \times C_{hs}^{\infty} \times exp^{\frac{-F\Psi_{dns}}{RT}} \times C_{cll}^{\infty} \times exp^{\frac{F\Psi_{dnl}}{RT}} \qquad S9.4$$

Under the standard condition, in the light phase, P_{k_1} and P_{k_2} was assigned the value of 4×10^{-8} cm s^{-1}; P_{cl_1} and P_{cl_2} was assigned the value of 8×10^{-8} cm s^{-1}; P_{h/k_1} and P_{h/k_2} was assigned the value of 10^6 cm^3 mol^{-1} s^{-1}; P_{h/cl_1} and P_{h/cl_2} was assigned the value of 10^6 cm^3 mol^{-1} s^{-1}. In the darkness, P_{k_1} and P_{k_2} was assigned the value of 1.2×10^{-8} cm s^{-1}; P_{cl_1} and P_{cl_2} was assigned the value of 1.2×10^{-4} cm s^{-1}; P_{h/k_1} and P_{h/k_2} was assigned the value of 10^9 cm^3 mol^{-1} s^{-1}; P_{h/cl_1} and P_{h/cl_2} was assigned the value of 10^6 cm^3 mol^{-1} s^{-1}.

X. Regulatory reactions

One of the regulatory pathways is Mehler type reaction which shunts electron from Fd^- to O_2, finally leading to formation of water. The equation describing this reaction can be summarized as follows:

$$V_{me} = k_{me}[O_2]_w \frac{[Fd^-]}{[Fd] + [Fd^-]} \qquad S10.1$$

Here, k_{me} is set to 4 s^{-1} (Laisk et al., 2006) (Laisk et al., 2006) and $[O_2]_w$ is O_2 in chloroplasts, the concentration corresponding to 21% O_2 in air and fixed as 0.265 mM (Laisk et al., 2006) (Laisk et al., 2006).

The model was incorporated with two main regulatory pathways induced by the lumenal acidification, which are non-photochemical quenching (NPQ) and 'back pressure' effect on the turnover of *Cytb₆f* oxidizing PQH_2. NPQ previously modeled by Ebenhöh et al. (2011) (Ebenhoh et al., 2011) was used here, which is calculated by

a differential equation:

$$\frac{d(NPQ)}{dt} = k_{npq\_f}(1 - NPQ)\frac{[H_i^+]^{n_{npq}}}{[H_i^+]^{n_{npq}} + \left(k_{npq\_eq}\right)^{n_{npq}}} - k_{npq_b}NPQ \qquad S10.2$$

Where n_{npq} (Hill coefficient) equals to 5, k_{npq_f} (forward rate constant) and k_{npq_b} (backward rate constant) are 0.05 s^{-1} and 0.004 s^{-1}, respectively, k_{npq_eq} denotes the dissociation constant of qE-active residues in LHCs of PSII with the value of 0.004 mmol $(mol\ chl)^{-1}$. All values were taken from Ebenhöh et al. (2011) (Ebenhoh et al., 2011). (1–NPQ) was multiplied by the rate constant of charge separation in PSII, k_{L2}, which reflects the rate of excitation formation in P680. This formalism is used to simply describe the energy dissipation in LHCs of PSII by the qE type of NPQ.

In the article by Van-Kooten et al. (1986) (Van Kooten et al., 1986), a factor k_{bp} was used to describe the 'back pressure' effect of lumenal acidification on the turnover of *Cytb$_6$f* oxidizing PQH_2, which can be written as follows:

$$\Delta\tilde{\mu}_{H^+} = \Delta\Psi_m F + 2.3RT\Delta pH \qquad S10.3$$

$$k_{bp} = \frac{1}{1 + co_{bp} \times \Delta\tilde{\mu}_{H^+}/(RT)} \qquad S10.4$$

Here, $\Delta\tilde{\mu}_{H^+}$ denotes the electrochemical potential of protons (J mol^{-1}) and CO_{bp} is taken to be 0.1 to obtain the best simulation.

XI. Solver

The above related set of differential and other equations, was coded in Matlab version 2018a (Mathworks Inc., USA). It was solved using a built-in solver (ode15s) which is competent to solve stiff ordinary differential equations with the assignment of initial values.

XII. References

Barber, J. (1982). Influence of Surface Charges on Thylakoid Structure and Function. *Annual Review of Plant Physiology* 33**,** 261-295.

Ebenhoh, O., Houwaart, T., Lokstein, H., Schlede, S., and Tirok, K. (2011). A minimal mathematical model of nonphotochemical quenching of chlorophyll fluorescence. *Biosystems* 103**,** 196-204.

Giersch, C., Heber, U., Kobayashi, Y., Inoue, Y., Shibata, K., and Heldt, H.W. (1980). Energy charge, phosphorylation potential and proton motive force in chloroplasts. *Biochim Biophys Acta* 590**,** 59-73.

Igamberdiev, A.U., and Kleczkowski, L.A. (2001). Implications of adenylate kinase-governed equilibrium of adenylates on contents of free magnesium in plant cells and compartments. *Biochem J* 360**,** 225-231.

Kinraide, T.B. (1994). Use of a Gouy-Chapman-Stern Model for Membrane-Surface Electrical Potential to Interpret Some Features of Mineral Rhizotoxicity. *Plant Physiol* 106**,** 1583-1592.

Kinraide, T.B., Yermiyahu, U., and Rytwo, G. (1998). Computation of surface electrical potentials of plant cell membranes . Correspondence To published zeta potentials from diverse plant sources. *Plant Physiol* 118**,** 505-512.

Kocks, P., and Ross, J. (1995). Kinetic Model for (Damped) Oscillations of Transthylakoid pH in Plants. *The Journal of Physical Chemistry* 99**,** 16490-16497.

Laisk, A., Eichelmann, H., and Oja, V. (2006). C3 photosynthesis in silico. *Photosynth Res* 90**,** 45-66.

Lazár, D. (2009). Modelling of light-induced chlorophyll a fluorescence rise (O-J-I-P transient) and changes in 820 nm-transmittance signal of photosynthesis. *Photosynthetica* 47**,** 483-498.

Lazar, D., and Jablonsky, J. (2009). On the approaches applied in formulation of a kinetic model of photosystem II: Different approaches lead to different simulations of the chlorophyll alpha fluorescence transients. *J Theor Biol* 257**,** 260-269.

Li, M., Svoboda, V., Davis, G., Kramer, D., Kunz, H.H., and Kirchhoff, H. (2021). Impact of ion fluxes across thylakoid membranes on photosynthetic electron transport and photoprotection. *Nat Plants* 7**,** 979-988.

Lyu, H., and Lazar, D. (2017a). Modeling the light-induced electric potential difference (DeltaPsi), the pH difference (DeltapH) and the proton motive force across the thylakoid membrane in C3 leaves. *J Theor Biol* 413**,** 11-23.

Lyu, H., and Lazar, D. (2017b). Modeling the light-induced electric potential difference DeltaPsi across the thylakoid membrane based on the transition state rate theory. *Biochim Biophys Acta Bioenerg* 1858**,** 239-248.

Muller, D.J., Dencher, N.A., Meier, T., Dimroth, P., Suda, K., Stahlberg, H., Engel, A., Seelert, H., and Matthey, U. (2001). ATP synthase: constrained stoichiometry of the transmembrane rotor. *FEBS Lett* 504**,** 219-222.

Ohshima, H. (2006). "Theory of Colloid and Interfacial Electric Phenomena," in *Theory of Colloid and Interfacial Electric Phenomena*. (San Diego: Elsevier Academic Press Inc), 1-473.

Pettersson, G. (1997). Control properties of the Calvin photosynthesis cycle at physiological carbon dioxide concentrations. *Biochimica et Biophysica Acta (BBA) - Bioenergetics* 1322**,** 173-182.

Pettersson, G., and Ryde-Pettersson, U. (1988). A mathematical model of the Calvin photosynthesis cycle. *Eur J Biochem* 175**,** 661-672.

Poolman, M.G., Assmus, H.E., and Fell, D.A. (2004). Applications of metabolic modelling to plant

metabolism. *J Exp Bot* 55**,** 1177-1186.

Poolman, M.G., Fell, D.A., and Thomas, S. (2000). Modelling photosynthesis and its control. *J Exp Bot* 51 Spec No**,** 319-328.

Sukhov, V., Akinchits, E., Katicheva, L., and Vodeneev, V. (2013). Simulation of variation potential in higher plant cells. *J Membr Biol* 246**,** 287-296.

Sukhov, V., Surova, L., Sherstneva, O., Bushueva, A., and Vodeneev, V. (2015). Variation potential induces decreased PSI damage and increased PSII damage under high external temperatures in pea. *Funct Plant Biol* 42**,** 727-736.

Sukhov, V., and Vodeneev, V. (2009). A mathematical model of action potential in cells of vascular plants. *J Membr Biol* 232**,** 59-67.

Van Kooten, O., Snel, J.F., and Vredenberg, W.J. (1986). Photosynthetic free energy transduction related to the electric potential changes across the thylakoid membrane. *Photosynth Res* 9**,** 211-227.

Zhu, X.G., Wang, Y., Ort, D.R., and Long, S.P. (2013). e-Photosynthesis: a comprehensive dynamic mechanistic model of C3 photosynthesis: from light capture to sucrose synthesis. *Plant Cell Environ* 36**,** 1711-1727.
